# Modeling the contrasting Neolithic male lineage expansions in Europe and Africa

**DOI:** 10.1186/2041-2223-4-25

**Published:** 2013-11-21

**Authors:** Michael J Sikora, Vincenza Colonna, Yali Xue, Chris Tyler-Smith

**Affiliations:** 1 The Wellcome Trust Sanger Institute, Wellcome Trust Genome Campus, Hinxton, Cambridgeshire CB10 1SA, UK; 2 Institute of Genetics and Biophysics, National Research Council (CNR), 80125, Naples, Italy

**Keywords:** Human Y chromosome, Neolithic transition, Population expansion, Demographic modeling, Coalescent simulations, Haplogroup, R1b, E1b1a

## Abstract

**Background:**

Patterns of genetic variation in a population carry information about the prehistory of the population, and for the human Y chromosome an especially informative phylogenetic tree has previously been constructed from fully-sequenced chromosomes. This revealed contrasting bifurcating and starlike phylogenies for the major lineages associated with the Neolithic expansions in sub-Saharan Africa and Western Europe, respectively.

**Results:**

We used coalescent simulations to investigate the range of demographic models most likely to produce the phylogenetic structures observed in Africa and Europe, assessing the starting and ending genetic effective population sizes, duration of the expansion, and time when expansion ended. The best-fitting models in Africa and Europe are very different. In Africa, the expansion took about 12 thousand years, ending very recently; it started from approximately 40 men and numbers expanded approximately 50-fold. In Europe, the expansion was much more rapid, taking only a few generations and occurring as soon as the major R1b lineage entered Europe; it started from just one to three men, whose numbers expanded more than a thousandfold.

**Conclusions:**

Although highly simplified, the demographic model we have used captures key elements of the differences between the male Neolithic expansions in Africa and Europe, and is consistent with archaeological findings.

## Background

Around 50 to 70 thousand years ago (approximately 60 KYA), modern humans expanded out of Africa, and by approximately 15 KYA had colonized all inhabitable continents [1]. During most of this period, the climate was both cold and unstable, but after approximately 10 KYA (the beginning of the Holocene period) it warmed and stabilized to produce the climate we know today. Early humans subsisted by hunting and gathering, but in the Holocene additional lifestyles became possible, including agriculture and pastoralism. This ‘Neolithic transition’ occurred independently at different times during the Holocene in different geographical regions. One Neolithic transition began in the Fertile Crescent in the Near East approximately 10 KYA and spread outwards in several directions, including into Europe over the course of several thousand years [2]. In sub-Saharan Africa, a comparable transition began later, approximately 3 KYA in West Africa, and spread south and east, reaching the extreme south only within historical times [3]. This differed from the transition in Europe in a number of respects: for example, there was no change in stone tool technology or use of copper or bronze, but instead a direct transition from the Later Stone Age to iron use, and some archaeologists therefore consider it inappropriate to use the term ‘Neolithic’ , but we retain it here because it is simple and widely understood. Both transitions were associated with large increases in population size.

Genetic evidence has contributed to our understanding of these events. There has been debate about the extent to which the genomes of present-day inhabitants of these areas have been derived from Neolithic farmers or from Paleolithic hunter-gatherers. The first large-scale molecular-genetic analyses in Europe were based on mitochondrial DNA (mtDNA) from present-day Europeans and were interpreted as favoring a Paleolithic entry for the majority of European mtDNAs [4]. More direct tests of this question, however, using ancient DNA (aDNA), have revealed a discontinuity between hunter-gatherer and early farmer mtDNAs, suggesting a Neolithic or later entry for the lineages that are most common today [5-8]. Similarly, low-coverage whole-genome sequencing supported the idea of a southern origin for early farmers from northern Europe [9,10], and thus migration and expansion of incoming Neolithic populations to replace the previous occupants.

The Y chromosome has several properties that make it potentially very informative about historical events, including the Neolithic transition. Its lack of recombination over most of its length means that it provides the most detailed and informative phylogenetic tree for any locus in the genome, while as a consequence of its strict father-to-son transmission it carries information specifically about male events [11]. Y-chromosomal lineages differ substantially between geographical regions and in each of the two areas considered here a single lineage predominates: R1b (especially the sublineage defined by the SNP M269, rs9786153) in Western Europe [12,13] and E1b1a (defined by the SNP known variously as M2, sY81, DYS271 or rs9785941) in sub-Saharan Africa [14]. While these observed geographical distributions are uncontested, and E1b1a has been widely associated with the Neolithic expansion in Africa [15,16], the time depth of R1b in Europe has been disputed, with opinions ranging from a Paleolithic date [13] to a Neolithic one [17]. aDNA has not yet been very informative for the Y chromosome, although the limited data available show no evidence of pre-Neolithic R1b lineages [5]. Full sequences from the Y chromosomes of present-day individuals, however, have recently become available, and these support a Neolithic spread of R1b [18]. In addition, the tree structure resulting from these sequences, based on the unbiased ascertainment of variants, is informative in other ways. There is a striking difference in the structure of the E1b1a and R1b phylogenies: R1b has a starlike structure indicative of an expansion so rapid that few mutations occurred during the expansion, while E1b1a has a more regular bifurcating structure.

In the current study, we accept R1b and E1b1a as lineages that expanded during the Neolithic, and set out to explore, using coalescent simulations, the demographic conditions under which their different phylogenetic structures might be expected to arise. We found that these differ between the two continents, and link our conclusions to the available archaeological evidence.

## Methods

### Data

The samples consisted of 21 high-coverage Y-chromosomal sequences downloaded from the Complete Genomics website [19], eight from the E1b1a haplogroup and 13 from the R1b haplogroup. Filtering of the data and generation of a phylogenetic tree from them have been described previously [18]. Eight individuals within the R1b haplogroup were from a three-generation pedigree, so in the current work where the simulations assume individuals are unrelated, this pedigree was combined to make a single branch by averaging the number of distinct SNPs in each family member and adding this value to the number of SNPs shared by all of the individuals.

### Coalescent simulations

Simulations were performed using MaCS [20], a coalescent simulator, using six and eight haplotypes for the R1b and E1b1a data, respectively, with a sequence length of 8.8 × 10^6^ nucleotides, assuming a generation time of 30 years [21], a mutation rate of 3 × 10^-8^ per nucleotide per generation [22] and zero recombination. The simulations explored the parameters of a single population expansion using four variables: the starting and final population sizes, the time when the expansion ended, and the length of the expansion. Examples of the command lines used are provided in Additional file 1: Table S2.

Since we needed to compare the output from the simulations with the trees from the real data, as described below, we constructed statistics related to ones used previously [23] to compare the output, as follows. The phylogenetic tree from each simulation was normalized to a total branch length of 1.0 and analyzed using three measures: the ratio of singletons to shared SNPs, and the mean and standard deviation of the TMRCA (Time to the Most Recent Common Ancestor) of all the individual haplotypes. The singleton/shared SNP ratio (*r*) was calculated by summing the terminal branch lengths and dividing by the sum of the internal branch lengths multiplied by one plus the sum of each internal branch length beneath its node:

$$r=\frac{\sum_{b=1}^{n_{\mathrm{TER}}}l_{b}}{\sum_{b=1}^{n_{\mathrm{INT}}}\left(l_{b•}\left(1+\sum_{\mathrm{bi}=1}^{n_{\mathrm{BEN}}}l_{\mathrm{bi}}\;\right)\right)}$$

where *b* is a tree branch of length *l*_*b*_, which has *n*_*BEN*_ branches of length *l*_*bi*_ beneath its node, *n*_*TER*_ is the number of terminal branches and *n*_*INT*_ is the number of internal branches.

The other two statistics were calculated by determining the branch length of the TMRCA of each combination of the individual haplotypes and computing the mean and standard deviation. The three statistics thus reflect both the time depth of the tree and how starlike its structure is.

### Comparison of data and coalescent simulations

To identify the range of simulation parameter values that best fit the empirical trees, we created heat maps of a summary value of the three statistics, designated the average normalized delta (AND) value. The AND value was computed by dividing the difference of the simulated statistic and the empirical statistic by the empirical statistic and averaging these three distances:

$$\mathrm{AND}=\frac{\left(r_{s}-r_{o}\right)/r_{o}+\left(m_{s}-m_{o}\right)/m_{o}+\left(d_{s}-d_{o}\right)/d_{o}}{3}$$

where the subscript *s* indicates a simulated value, *o* an observed value, *r* a singleton/shared ratio statistic, *m* a mean TMRCA statistic and *d* a standard deviation of a TMRCA statistic.

A low AND value thus indicates a good fit to the empirical data. We completed 1,000 simulations for each demographic scenario and averaged each statistic to use as the simulated value.

The ranges for the parameters on the first set of simulations and corresponding heat map were each chosen to be very wide, including all reasonable estimates for their values (Additional file 2: Table S1). The parameter ranges for the time the expansion ended and the length of the expansion were each extended past the empirical TMRCA for each respective haplogroup. For each successive heat map, a conservative selection of the lowest AND values was noted and the ranges for the following set of simulations chosen to include these, unless their TMRCAs were not compatible with the maximum TMRCA of the haplogroup. Thus we sequentially removed parameter values that resulted in large AND values, progressively narrowing the range until it encompassed only AND values of 0.05 and below. Although these do not provide an absolute measure of how well the model fits the data, they show that among the wide ranges of parameters explored, these are the best fits. Then, a histogram for each parameter was created using the frequency of sub-0.05 AND values, to provide an indication of our conclusions regarding this parameter value.

## Results

**Figure 1:**
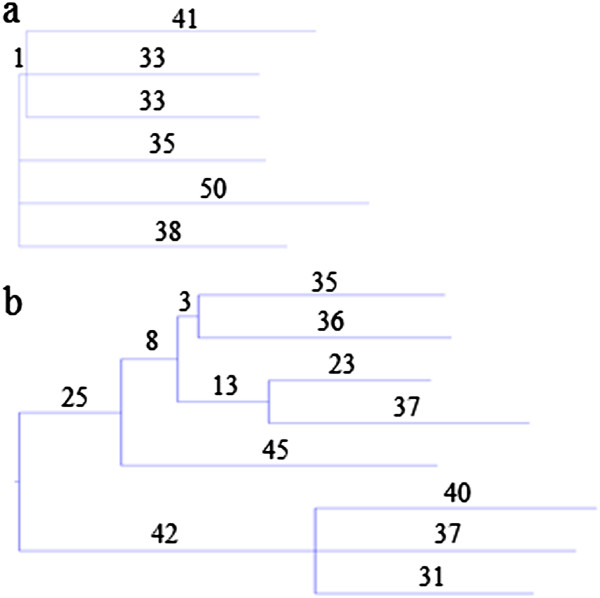
**Phylogenies based on high-coverage whole-genome sequences. (a)** Six R1b and **(b)** eight E1b1a Y chromosomes. Branch lengths are proportional to the number of SNPs, which are given on each branch, and thus approximately proportional to time.

The phylogenetic trees of the R1b and E1b1a branches of the Y-chromosomal phylogeny show strongly contrasting structures (Figure 1), as previously noted [18]. R1b has a markedly starlike structure (Figure 1a), with only a single variant uniting three of the six chromosomes creating a departure from a perfect star, while E1b1a shows a largely bifurcating structure with greater time depth and just one trifurcation (Figure 1b).

**Figure 2:**
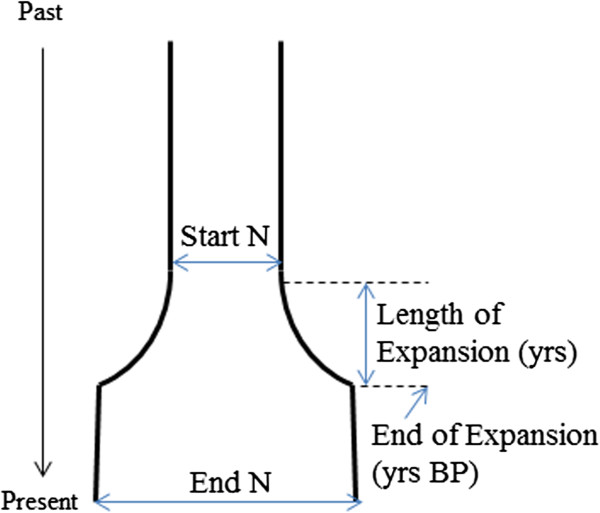
**Demographic model used in coalescent simulations.** A single exponential expansion was modeled, with four variable parameters as shown.

**Figure 3:**
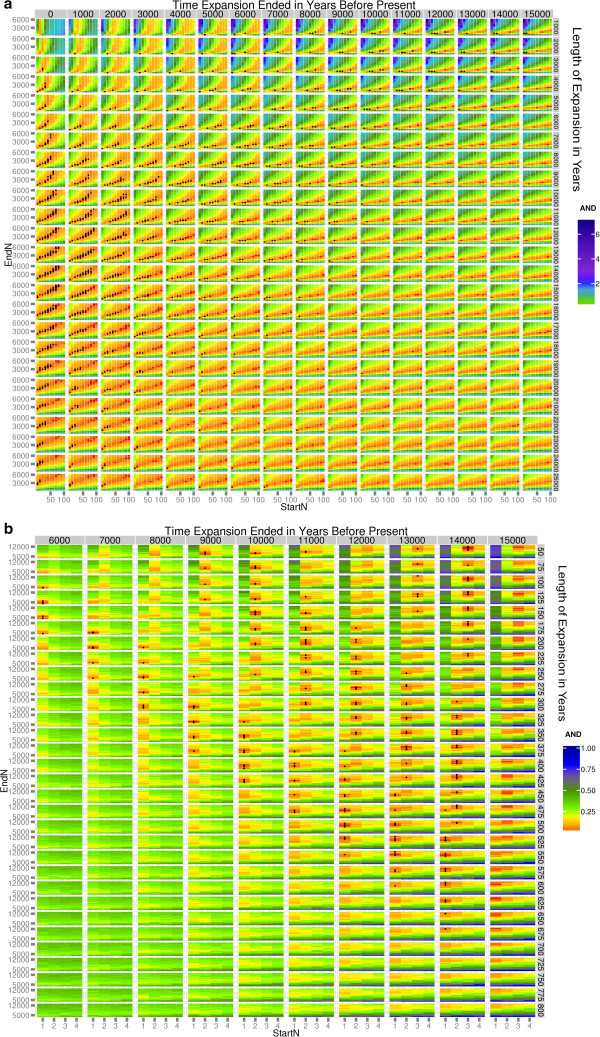
**Fit between model and observed data.** The color of the small rectangles indicates the AND value, which measures the fit between the model and the observed tree. Red: good fit, yellow and green: intermediate fits, blue: poor fit, as indicated by the scale. Each rectangle is based on 1,000 simulations. The best-fitting rectangles (AND < 0.05) are marked with black dots. AND, average normalized delta.

**Figure 4:**
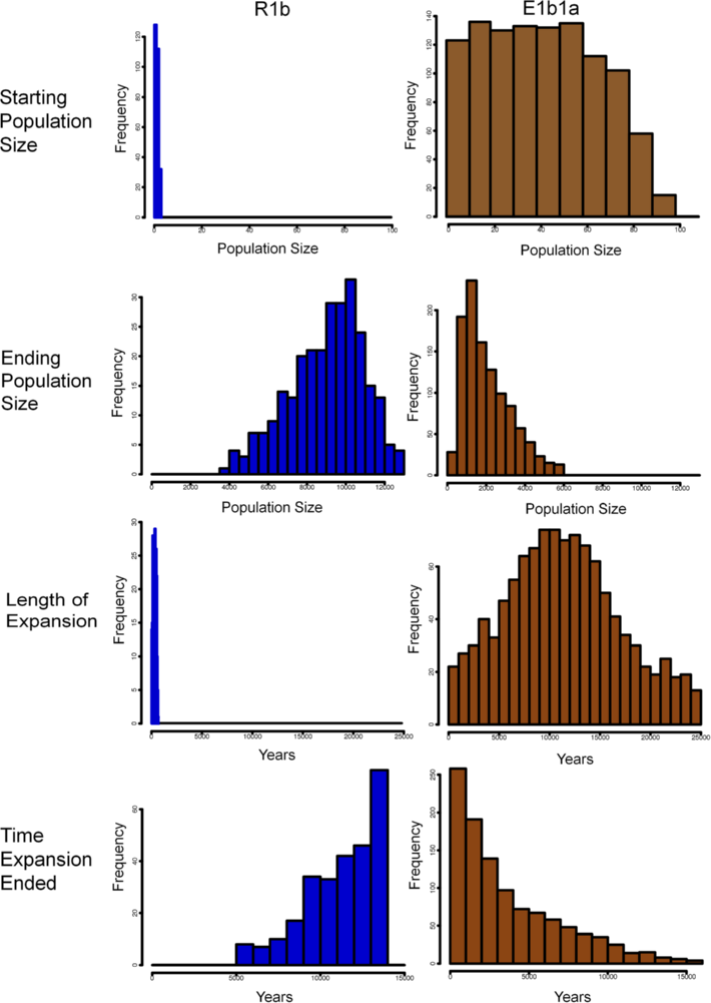
**Best-fitting parameter values.** Distributions of values for the four parameters from the simulations that fitted the empirical data best (AND < 0.05).

To explore demographic scenarios that could lead to these different structures, we performed coalescent simulations that included four parameters: starting and ending population sizes, and length and end time of the expansion (Figure 2). We used a strategy of sequential rounds of simulations, starting with a broad range of parameter values, assessing which combinations of these led to the best fit with the observed data, and then repeating the simulations with a narrower range of values centered around those that led to the best fit. These results are presented visually as heat maps illustrating the AND values, which measure the simulation-observed match (Figure 3 and Additional file 3: Figures S1-S14). In these heat maps, the color of the small rectangles indicates the AND value: red is for a good fit, yellow and green are for intermediate fits and blue is for a poor fit, as in the scale on the right of the maps. These small rectangles are assembled into sets with differing values of the starting population size (StartN, bottom) and ending population size (EndN, left) to form a grid of intermediate-sized rectangles separated by grey/white borders. These grids have different times for when the expansion ended (top) and different expansion lengths (right). The best-fitting small rectangles in Figure 3 (AND < 0.05) are marked with black dots. After 9 and 11 rounds of simulations for R1b and E1b1a, respectively, we obtained simulation sets in which a substantial proportion of the parameter combinations showed a good fit between the simulations and the observed data, indicated by an AND value of <0.05. We summarize the distribution of individual parameter values from these well-fitting simulations in Figure 4.

**Figure 5:**
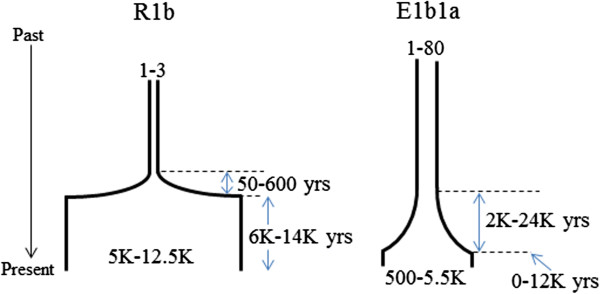
Favored demographic models for the European and African Neolithic expansions.

The simulations suggest that very different demographic histories are needed to generate the R1b and E1b1a trees. In Europe, the expansion in size was extreme, from a starting size of just two men (range one to three; numbers are given as the median and 95% interval from the data in Figure 4, rounded appropriately) to an ending size of approximately 9,500 (5,000 to 12,500), while in Africa it was extensive but less extreme, from a starting size of approximately 40 (1 to 80) to an ending size of approximately 2,000 (500 to 5,500). In Europe, the expansion was very rapid, taking only approximately 325 (50 to 600) years and ending approximately 12 (6 to 14) KYA, while in Africa it was considerably less rapid, taking approximately 12 (2 to 24) KY and ending more recently, approximately 2 (0 to 12) KYA. The resulting most favored scenarios are illustrated in Figure 5.

## Discussion

The model we have explored, involving a single exponential expansion, is grossly simplified. In addition, we have analyzed within each population a single lineage (R1b or E1b1a) of a single locus (the Y chromosome), and this may not be representative of the population. Nevertheless, there are several reasons to believe that our results should capture features of interest. First, the male history represented by the Y chromosome is of interest whether or not it corresponds to the history of other regions of the genome. Second, the single Y lineages we examined are the most frequent in their respective geographical regions, being found in >75% and >80% of males from many Western European and sub-Saharan African populations, respectively, so form a major constituent of the Y-chromosomal gene pool. Furthermore, the chromosomes sampled within each of the two lineages have diverse geographical origins: the R1b chromosomes come from the CEU (Northwestern Europe [24]), TSI (Italy), PUR and MXL (probably Iberia) populations, while the E1b1a chromosomes come from the YRI (Nigeria), LWK (Kenya) and ASW (probably West Africa) populations. Thus their origins are not confined to any one country or small geographical area, and are likely to be broadly representative of these lineages. Third, the Y phylogenies, based on resequencing approximately 9 Mb of Y-chromosomal DNA, are very robust, especially in this high-coverage dataset where singletons will be called reliably. Consequently the R1b chromosomes in this set, for example, must have radiated in an interval so short that there was only enough time for a single mutation to occur, no matter how complex the migrations, integrations or replacements and other cultural changes going on in the society carrying these chromosomes. Fourth, although only a portion of the parameter space has been explored within the model, and it remains possible (indeed, it is an inevitable feature of this approach), that an undiscovered global optimum with very narrow parameter values may exist, our sequential approach (Additional files 3: Figures S1 to S14) minimizes the chance of this, and we discuss below the good correspondence with other sources of information.

With these caveats, we can consider how the Y-chromosome-based genetic findings fit with other genetic and archaeological evidence. The Neolithic transition in Europe has been studied extensively by archaeologists. It appeared in Greece approximately 9 KYA and reached the extreme west by approximately 4 KYA [1,2]. The demographic model suggests that the R1b expansion most likely ended before this time, at approximately 12 KYA (Figures 4 and 5), which appears inconsistent with a Neolithic expansion of this lineage, although the lower limit does extend to approximately 6 KYA. We interpret the discrepancy, however, as a limitation of the model. We constrained the parameter values so that R1b could not expand before the estimated TMRCA of the sampled R1b chromosomes [18], and the model favored an immediate expansion of the lineage, hence the expansion at approximately 12 KYA. If we had used the more likely 4 to 5 KYA estimate of the R1b TMRCA from the rho statistic [18], the expansion in the current model would have been placed close to this time, well within the Neolithic and, interestingly, also close to the time of establishment of the major European mtDNA haplogroup, H, approximately 6 KYA [7,8]. The rapidity of the R1b expansion and the large increase in population size are most consistent with migration and population replacement, issues debated by archaeologists but favored by the aDNA data [5-9]. The later and more gradual E1b1a expansion in Africa is as expected from the spread of cattle-herders from the north between 2.5 and 8 KYA, followed by the Bantu expansion to the southern tip of the continent beginning approximately 2.5 KYA and ending within the last few hundred years, incorporating the package of Bantu languages, cattle and iron-working [1,3]. The population sizes used by the model are genetic effective population sizes, which, for a population that has expanded recently, are much smaller than the census population size [1].

Studies of this kind can be improved by considering more complex demographic models and larger Y-chromosomal datasets. While it may seem obvious that more complex and thus more realistic models should be preferable, models are only useful if the different scenarios they encompass can be discriminated between using the data available, so the simplest model that captures a relevant aspect of the data may still be the most appropriate one. Thus while future models in this context could incorporate spatial structure and phenomena such as surfing [25], a single rapid expansion should still be permitted. We have modeled only a single Y haplogroup, because in each expansion a single haplogroup predominates. Low-coverage sequencing of larger population samples by the 1000 Genomes Project [26,27] and two recent studies focusing on Africa [28] and Sardinia [29] confirm both the high frequencies of haplogroups R1b and E1b1a in the relevant populations and the structures of the phylogenetic trees associated with them. These projects thus provide much larger datasets, which could be used in future modeling studies, although the low coverage and substantial false negative rates of rare variants would need to be taken into account. With such data, the additional rare Y haplogroups present in the populations could also be considered. Different studies have come to different conclusions about the Y-chromosomal mutation rate [22,28,29]; in the current study, the mutation rate is used simply to scale the results, and a mutation rate about half [29] of that used here [22], for example, would double the times. Finally, we note that such analyses of single lineages, which may have deep coalescences, contrast with the universal sharing of recent genealogical ancestors by all people within the last few thousand years [30].

## Conclusions

We have identified demographic scenarios that can lead to the contrasting phylogenies observed for the major Y-chromosomal lineages that expanded during the distinct Neolithic transitions in Europe and Africa. These suggest that in Europe, the R1b lineage experienced an extremely rapid and extensive increase as soon as it entered the continent, expanding more than a thousandfold in a few generations. The expansion in Africa began from a larger population size, took thousands of years and ended only recently. While these conclusions are based on a simplified demographic model, they capture major differences between the continents and fit many aspects of the archaeological findings.

## Abbreviations

aDNA: Ancient DNA; AND: Average normalized delta; KYA: Thousand years ago; mtDNA: Mitochondrial DNA; SNP: Single nucleotide polymorphism; TMRCA: Time to the most recent common ancestor.

## Competing interests

The authors declare that they have no competing interests.

## Authors’ contributions

CTS and YX conceived the study. MJS, VC, YX and CTS designed the study. MJS performed the simulations. MJS, VC, YX and CTS interpreted the results. MJS and CTS drafted the manuscript. All authors read and approved the final manuscript.

## Supplementary Material

Additional file 1: Table S2 Examples of commands for MaCS. An example of a command for each of the R1b and E1b1a simulations is shown, along with the parameter set from which the command was derived. Click here for file

Additional file 2: Table S1 Starting parameter values for the simulations. Click here for file

Additional file 3: Figures S1 to S14 Heat maps illustrating the AND values from sequential simulation runs. Click here for file
