# Supplementary material for: Modeling the contrasting Neolithic male lineage expansions in Europe and Africa
Source: Investig Genet. 2013 Nov 21;4:25. doi: 10.1186/2041-2223-4-25 (PMC4177147; doi:10.1186/2041-2223-4-25)
Supplement: Additional file 1: Table S2 — Examples of commands for MaCS. An example of a command for each of the R1b and E1b1a simulations is shown, along with the parameter set from which the command was derived. [file 2041-2223-4-25-S1.doc]

Supplementary Table 2. Examples of commands for MaCS. An example of a command for each of the R1b and E1b1a simulations is shown, along with the parameter set from which the command was derived.

| R1b | macs 6 8.8e6 -T -t 3e-8 -G 57646 –eG 1.4e-4 0 | |
| --- | --- | --- |
| E1b1a | macs 8 8.8e6 -T -t 3e-8 -eG 2.1e-3 420 –eG 0.0125 0 | |
|  | | |
|  | R1b | E1b1a |
| Starting Population Size | 2 | 50 |
| Ending Population Size | 6,000 | 4,000 |
| Length of Expansion (yrs) | 100 | 5,000 |
| End of Expansion (yrs BP) | 0 | 1,000 |
