# Supplementary material for: Modeling the contrasting Neolithic male lineage expansions in Europe and Africa
Source: Investig Genet. 2013 Nov 21;4:25. doi: 10.1186/2041-2223-4-25 (PMC4177147; doi:10.1186/2041-2223-4-25)
Supplement: Additional file 2: Table S1 — Starting parameter values for the simulations. [file 2041-2223-4-25-S2.doc]

Supplementary Table 1. Starting parameter values for the simulations

|  | **R1b** | **E1b1a** |
| --- | --- | --- |
| Starting Population Size | 1-500 | 1-1,000 |
| Ending Population Size | 1,000-800,000 | 250-200,000 |
| Length of Expansion (yrs) | 50-20,000 | 100-50,000 |
| End of Expansion (yrs BP) | 1,000-20,000 | 0-50,000 |
