# Supplementary material for: Modeling the contrasting Neolithic male lineage expansions in Europe and Africa
Source: Investig Genet. 2013 Nov 21;4:25. doi: 10.1186/2041-2223-4-25 (PMC4177147; doi:10.1186/2041-2223-4-25)
Supplement: Additional file 3: Figures S1 to S14 — Heat maps illustrating the AND values from sequential simulation runs. [file 2041-2223-4-25-S3.pptx]

## Slide 1
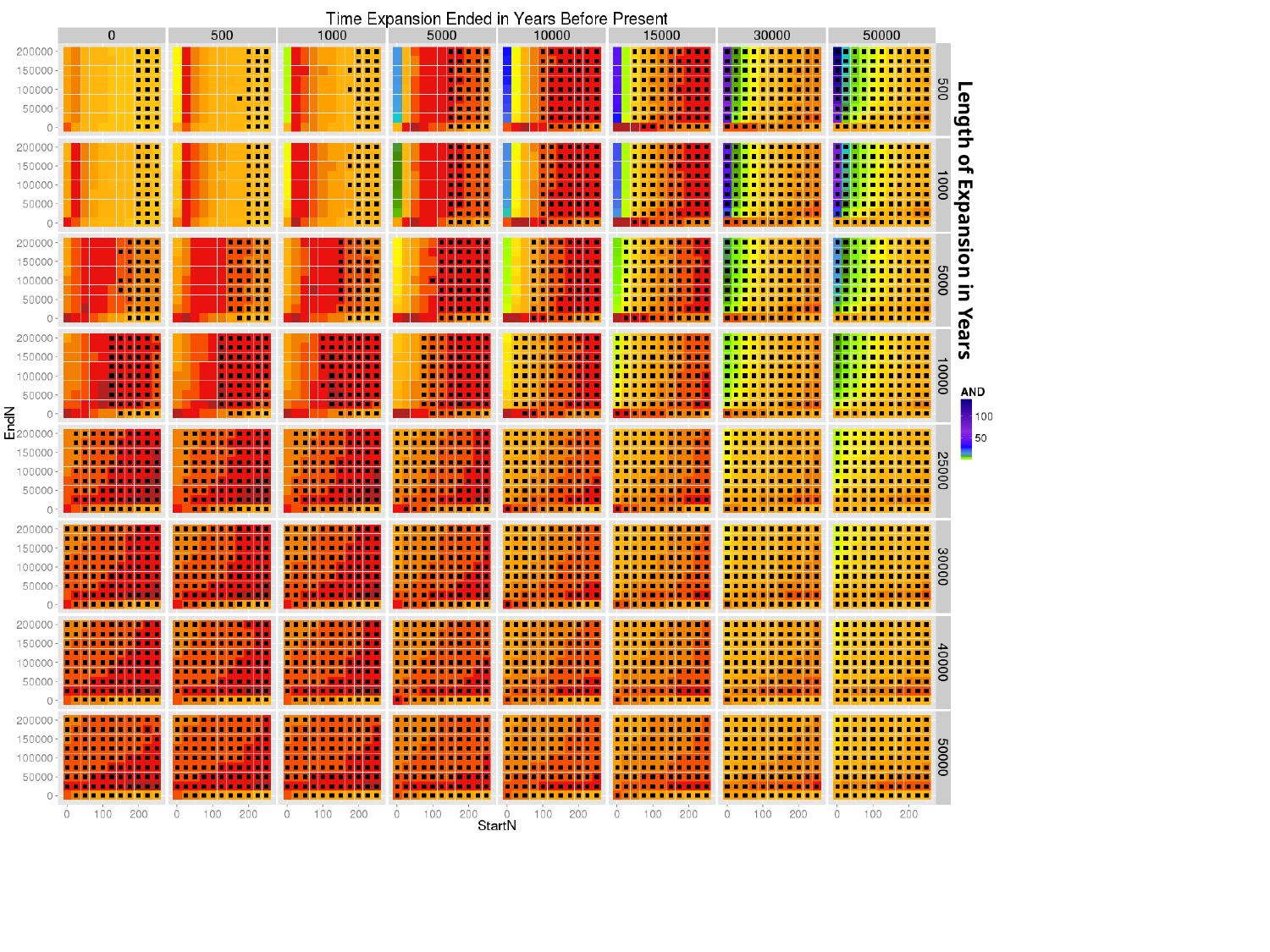

Length of Expansion in Years

## Slide 2
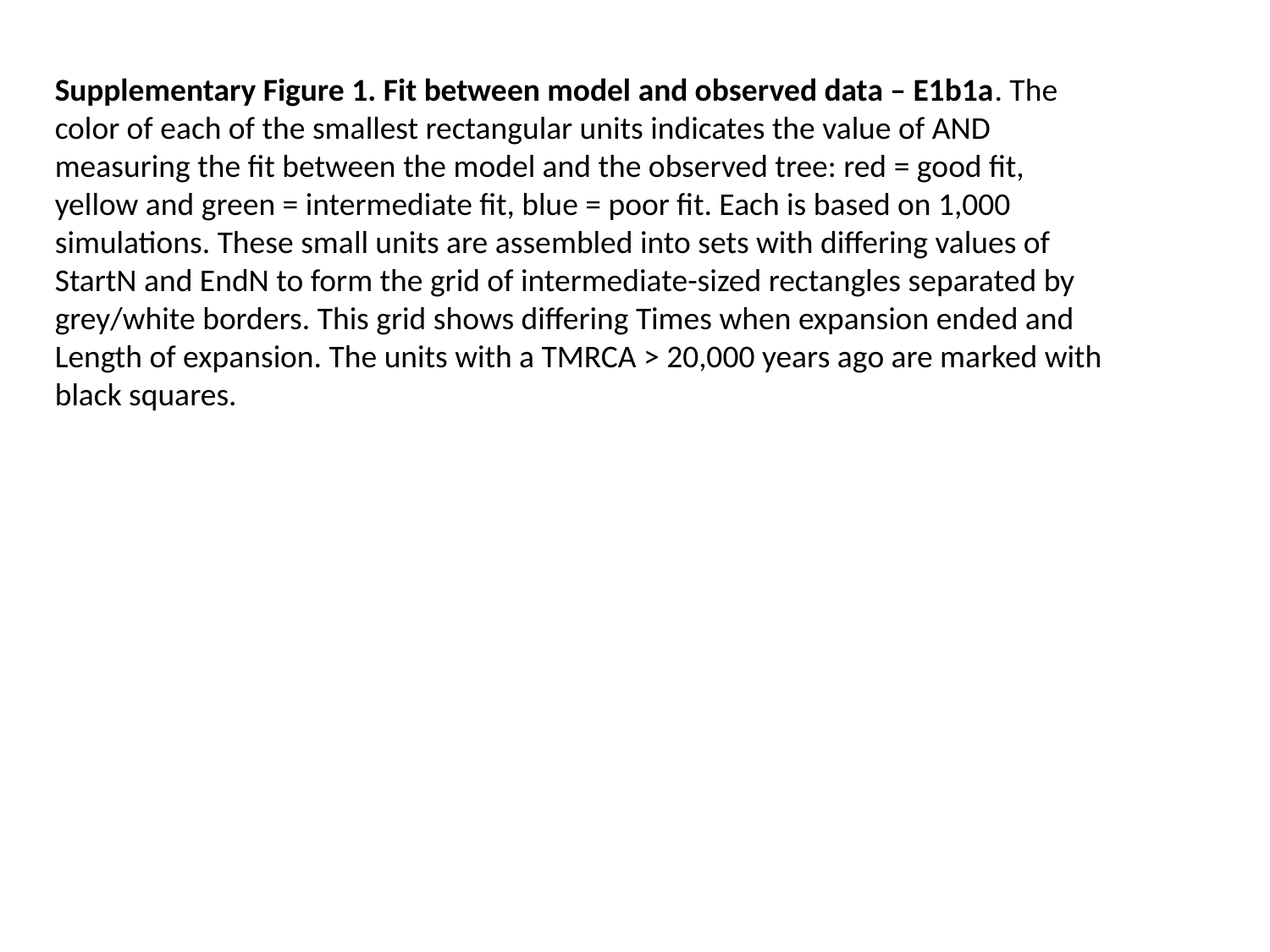

Supplementary Figure 1. Fit between model and observed data – E1b1a. The color of each of the smallest rectangular units indicates the value of AND measuring the fit between the model and the observed tree: red = good fit, yellow and green = intermediate fit, blue = poor fit. Each is based on 1,000 simulations. These small units are assembled into sets with differing values of StartN and EndN to form the grid of intermediate-sized rectangles separated by grey/white borders. This grid shows differing Times when expansion ended and Length of expansion. The units with a TMRCA > 20,000 years ago are marked with black squares.

## Slide 3
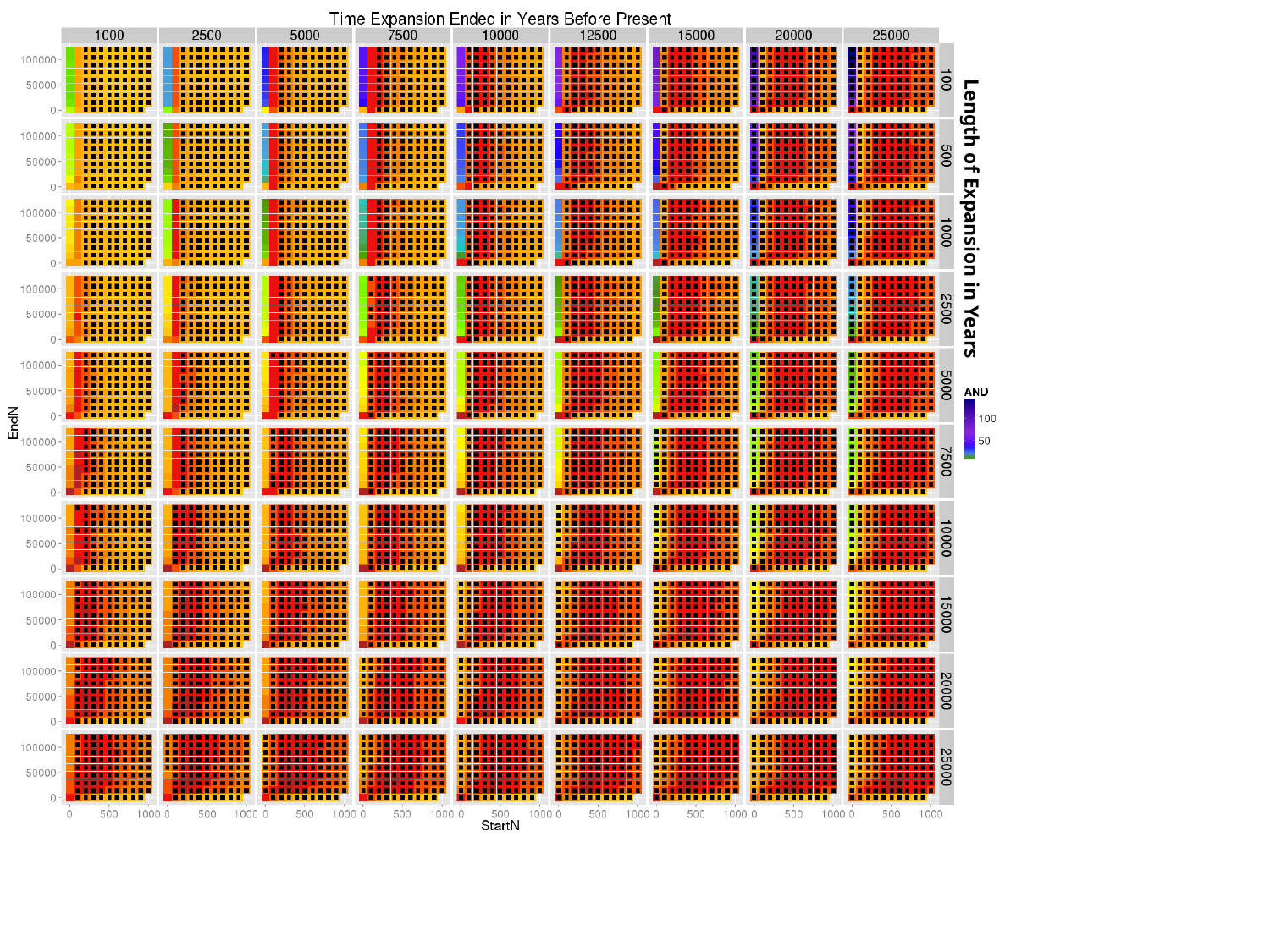

Length of Expansion in Years

## Slide 4
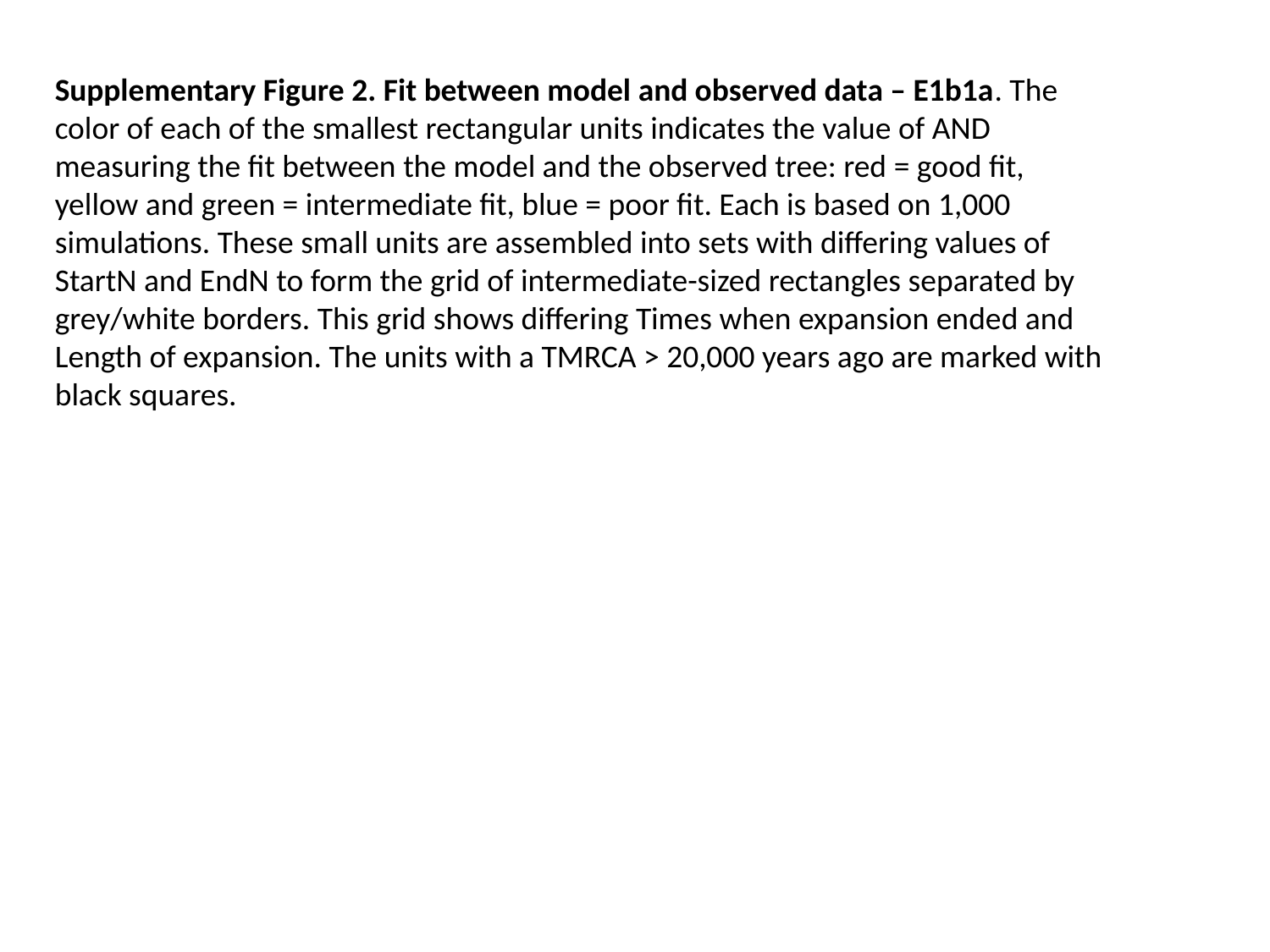

Supplementary Figure 2. Fit between model and observed data – E1b1a. The color of each of the smallest rectangular units indicates the value of AND measuring the fit between the model and the observed tree: red = good fit, yellow and green = intermediate fit, blue = poor fit. Each is based on 1,000 simulations. These small units are assembled into sets with differing values of StartN and EndN to form the grid of intermediate-sized rectangles separated by grey/white borders. This grid shows differing Times when expansion ended and Length of expansion. The units with a TMRCA > 20,000 years ago are marked with black squares.

## Slide 5
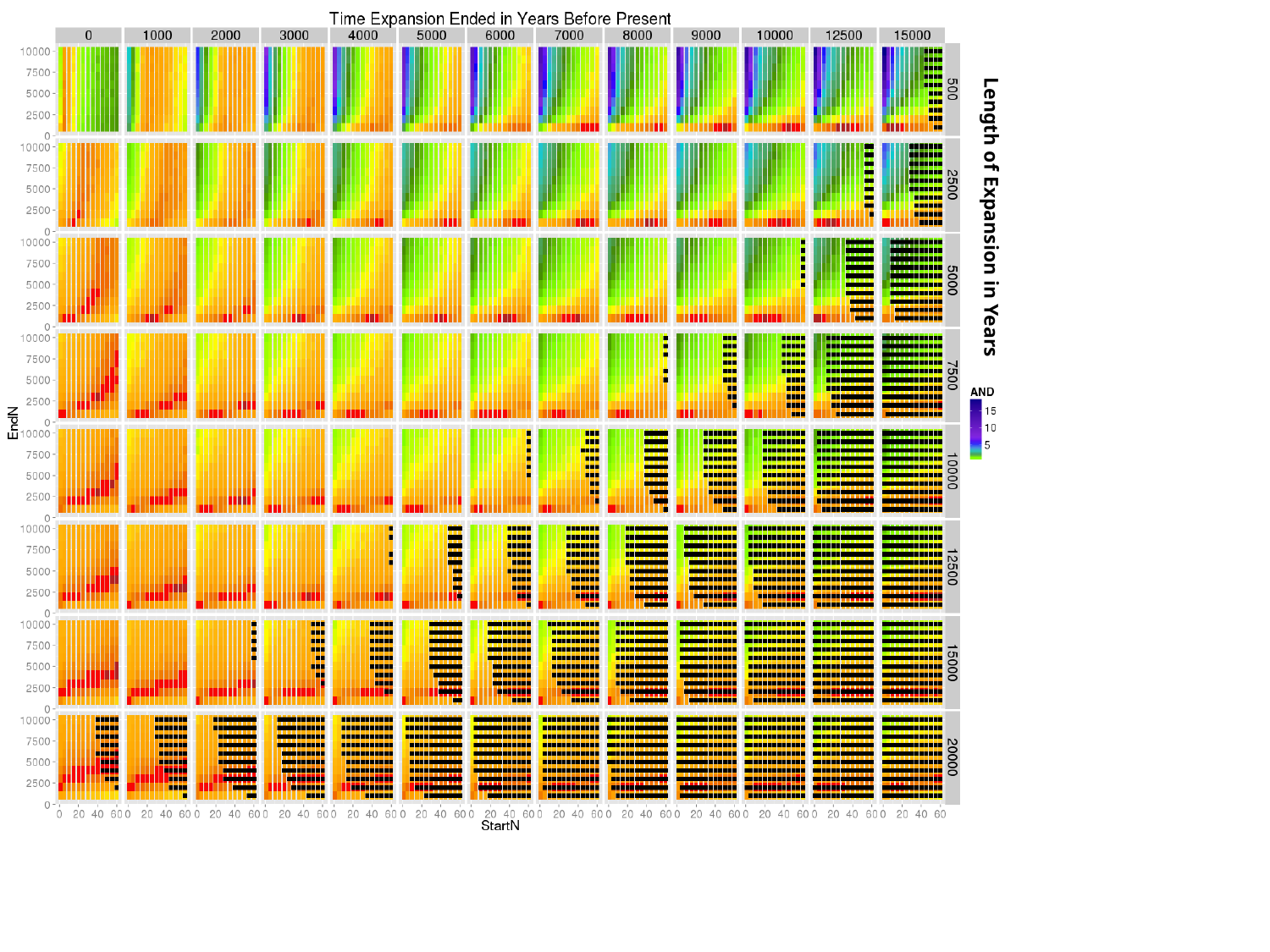

Length of Expansion in Years

## Slide 6
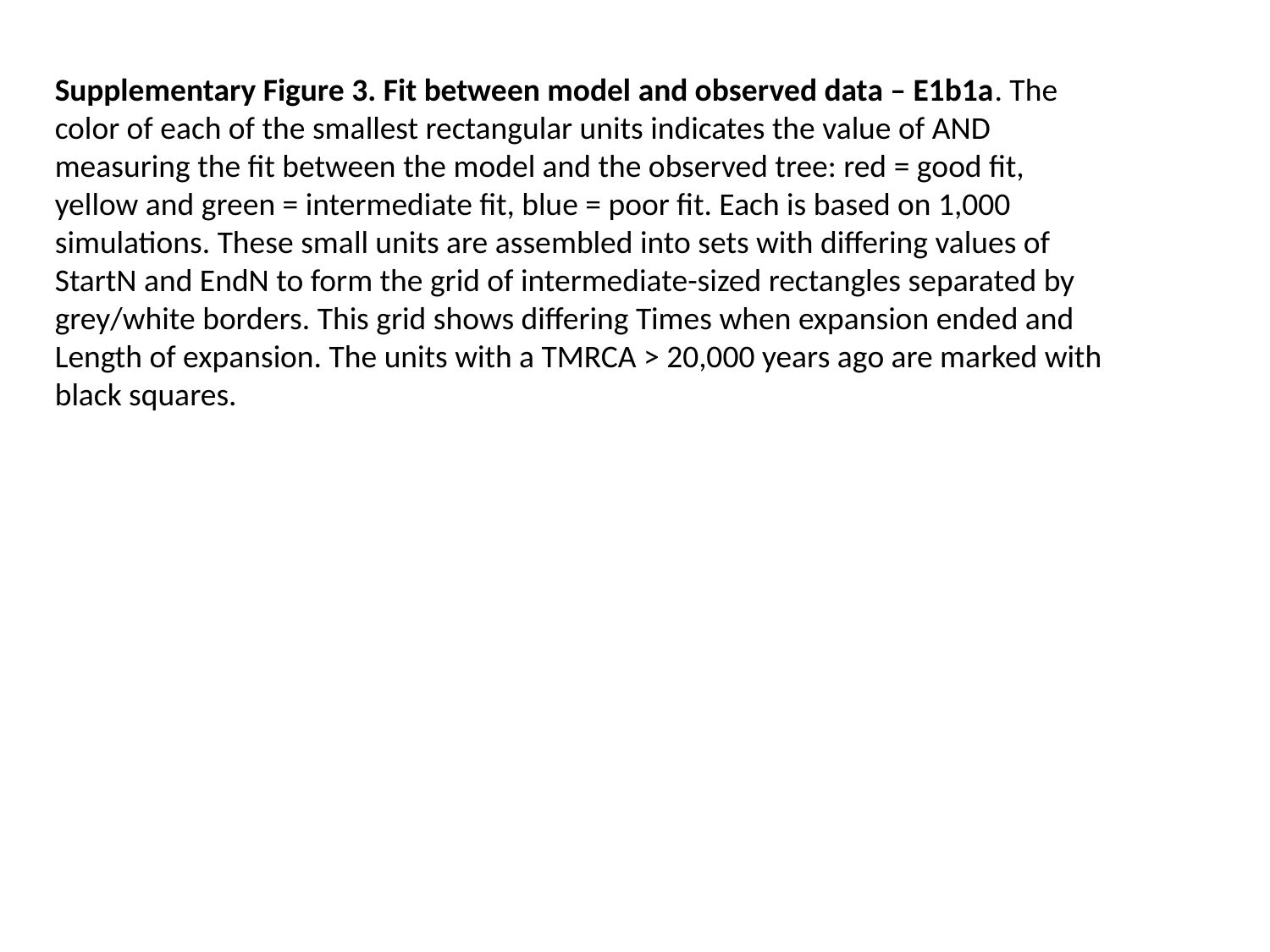

Supplementary Figure 3. Fit between model and observed data – E1b1a. The color of each of the smallest rectangular units indicates the value of AND measuring the fit between the model and the observed tree: red = good fit, yellow and green = intermediate fit, blue = poor fit. Each is based on 1,000 simulations. These small units are assembled into sets with differing values of StartN and EndN to form the grid of intermediate-sized rectangles separated by grey/white borders. This grid shows differing Times when expansion ended and Length of expansion. The units with a TMRCA > 20,000 years ago are marked with black squares.

## Slide 7
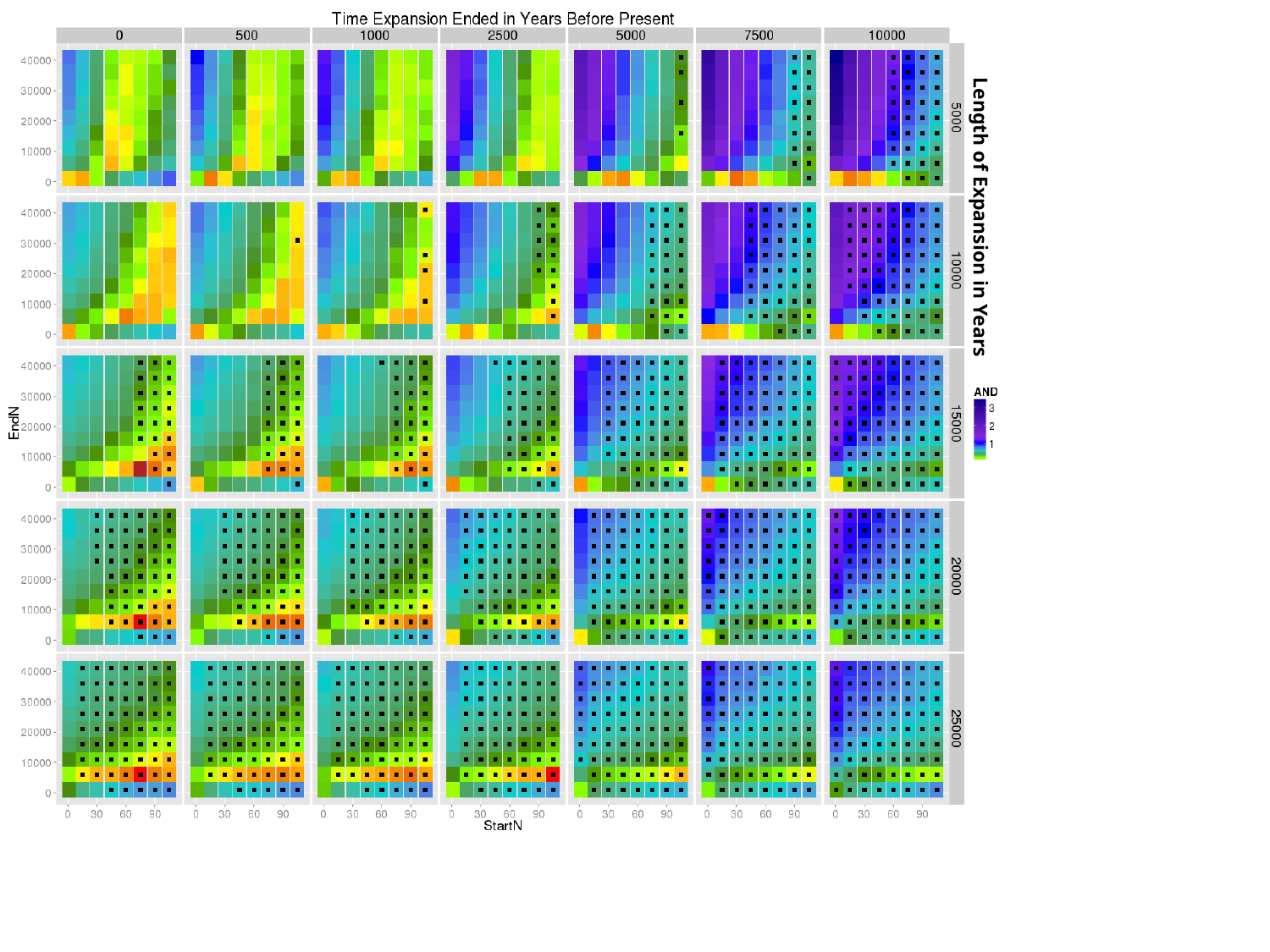

Length of Expansion in Years

## Slide 8
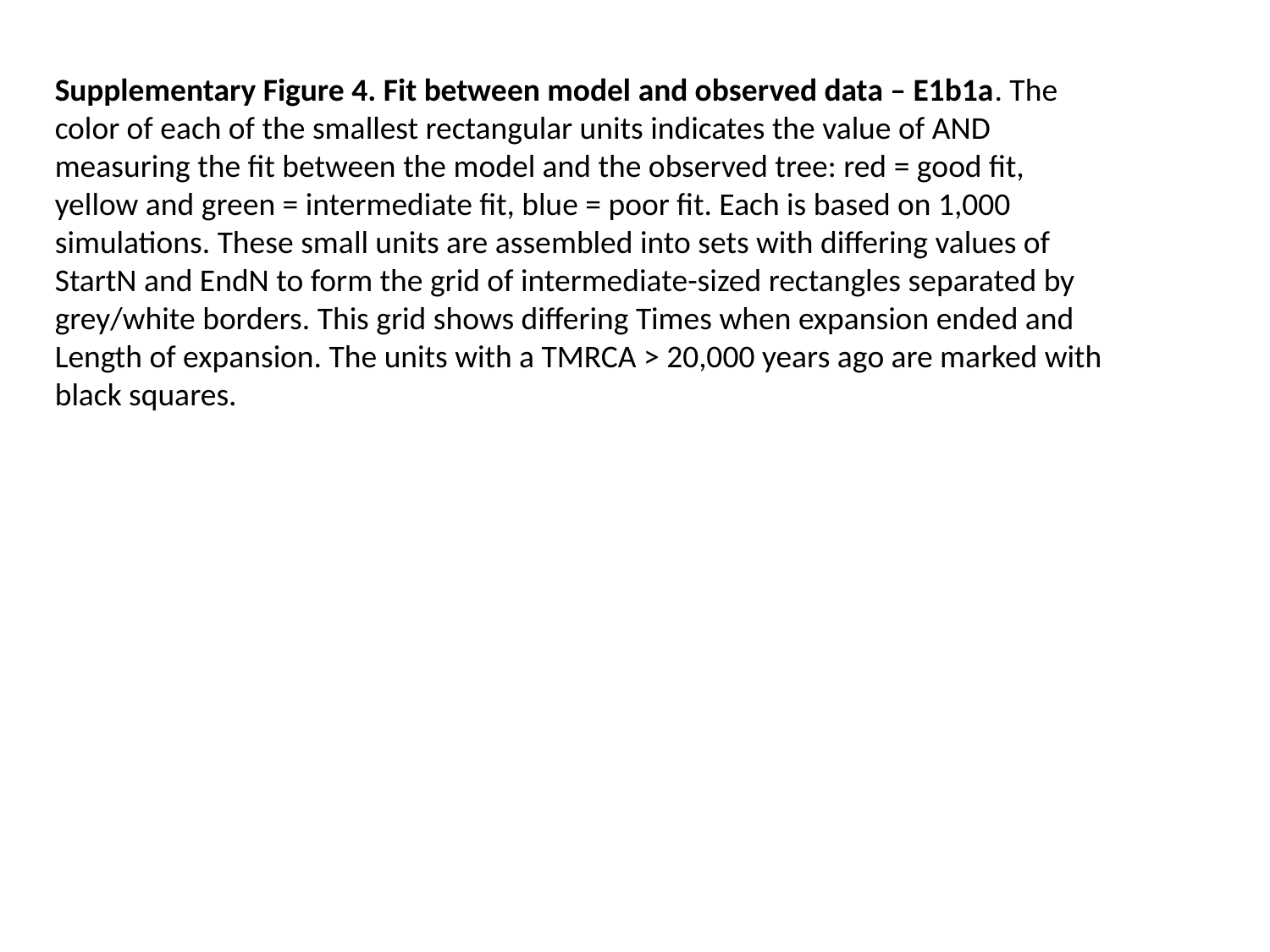

Supplementary Figure 4. Fit between model and observed data – E1b1a. The color of each of the smallest rectangular units indicates the value of AND measuring the fit between the model and the observed tree: red = good fit, yellow and green = intermediate fit, blue = poor fit. Each is based on 1,000 simulations. These small units are assembled into sets with differing values of StartN and EndN to form the grid of intermediate-sized rectangles separated by grey/white borders. This grid shows differing Times when expansion ended and Length of expansion. The units with a TMRCA > 20,000 years ago are marked with black squares.

## Slide 9
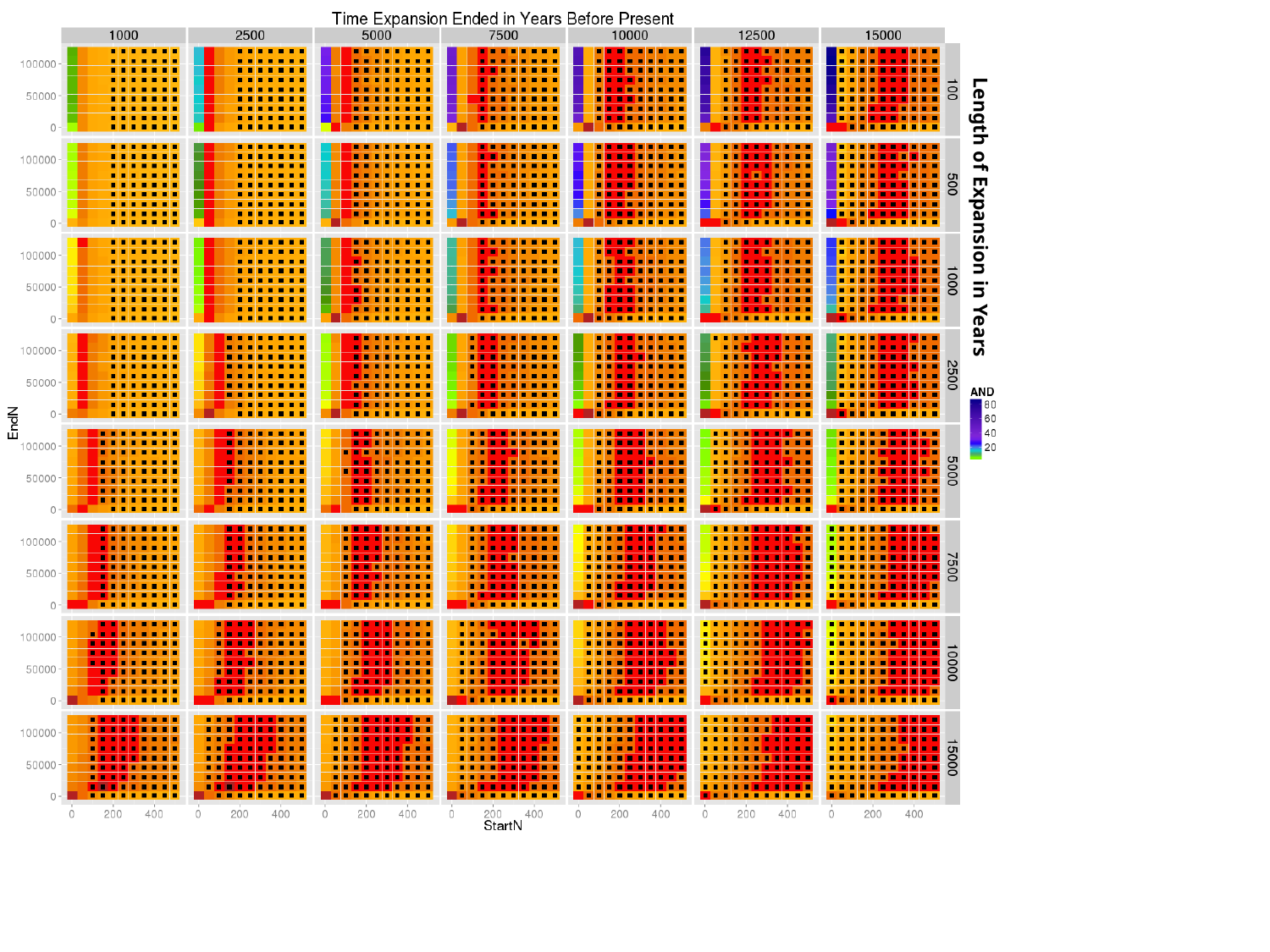

Length of Expansion in Years

## Slide 10
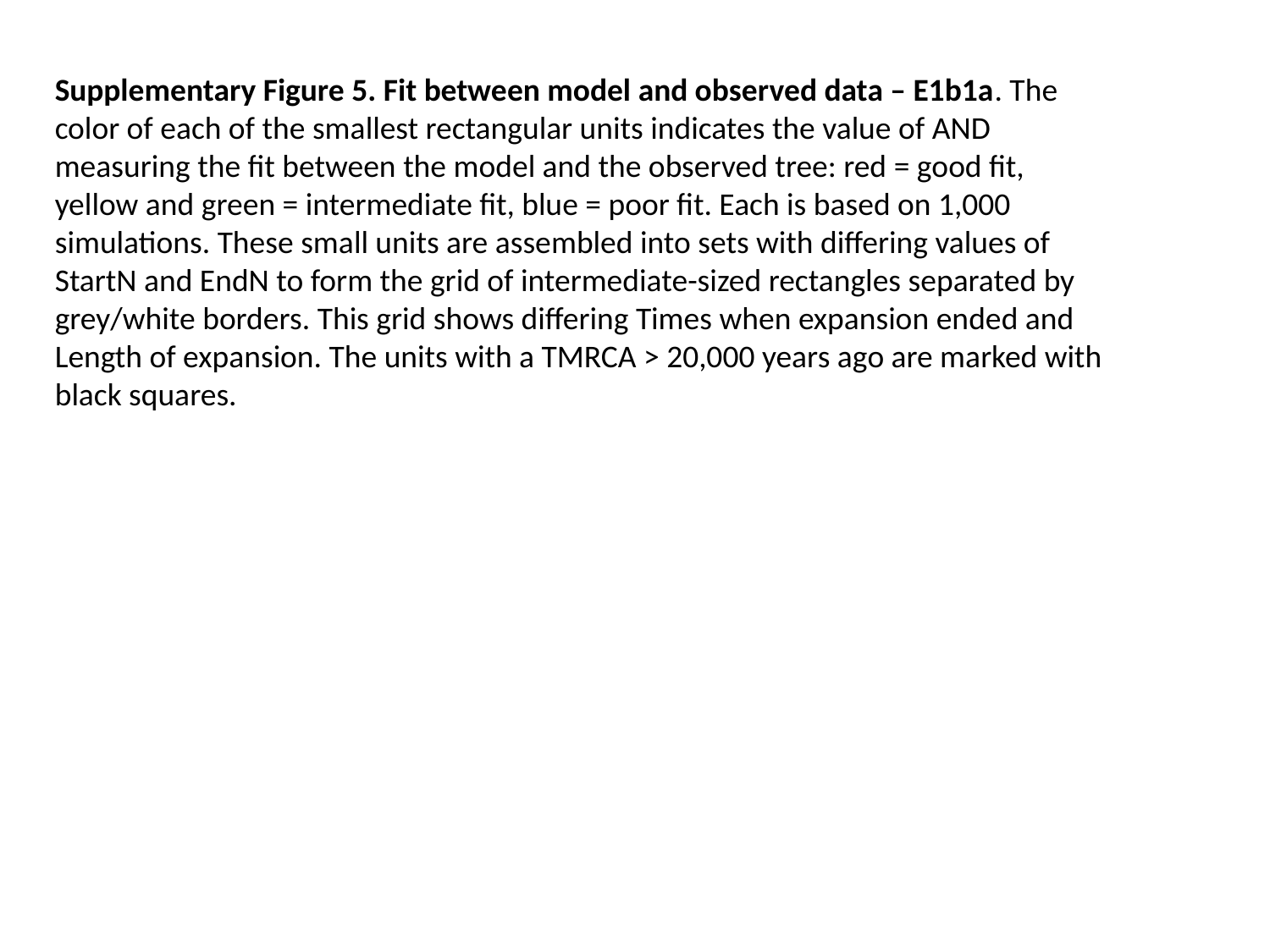

Supplementary Figure 5. Fit between model and observed data – E1b1a. The color of each of the smallest rectangular units indicates the value of AND measuring the fit between the model and the observed tree: red = good fit, yellow and green = intermediate fit, blue = poor fit. Each is based on 1,000 simulations. These small units are assembled into sets with differing values of StartN and EndN to form the grid of intermediate-sized rectangles separated by grey/white borders. This grid shows differing Times when expansion ended and Length of expansion. The units with a TMRCA > 20,000 years ago are marked with black squares.

## Slide 11
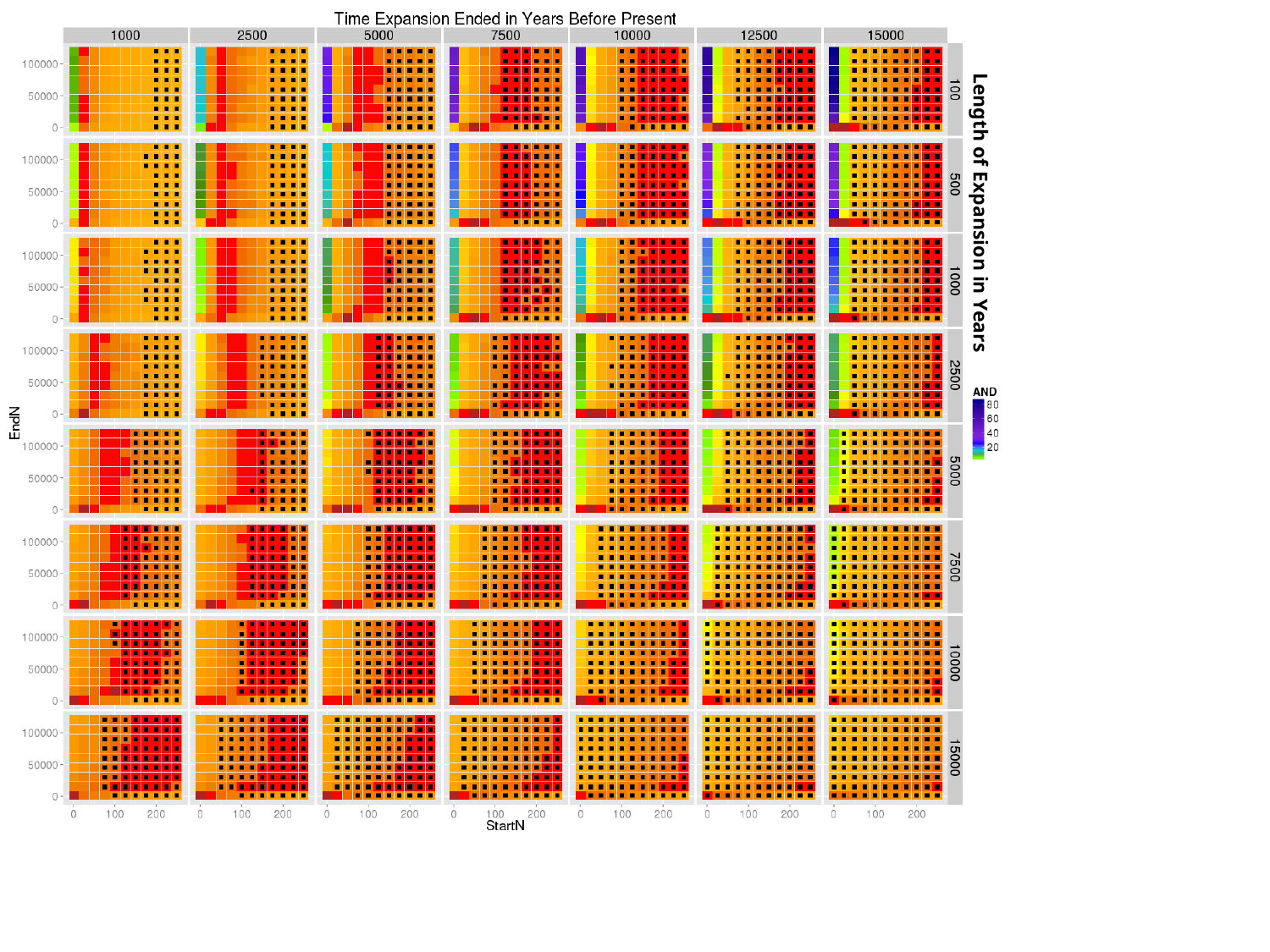

Length of Expansion in Years

## Slide 12
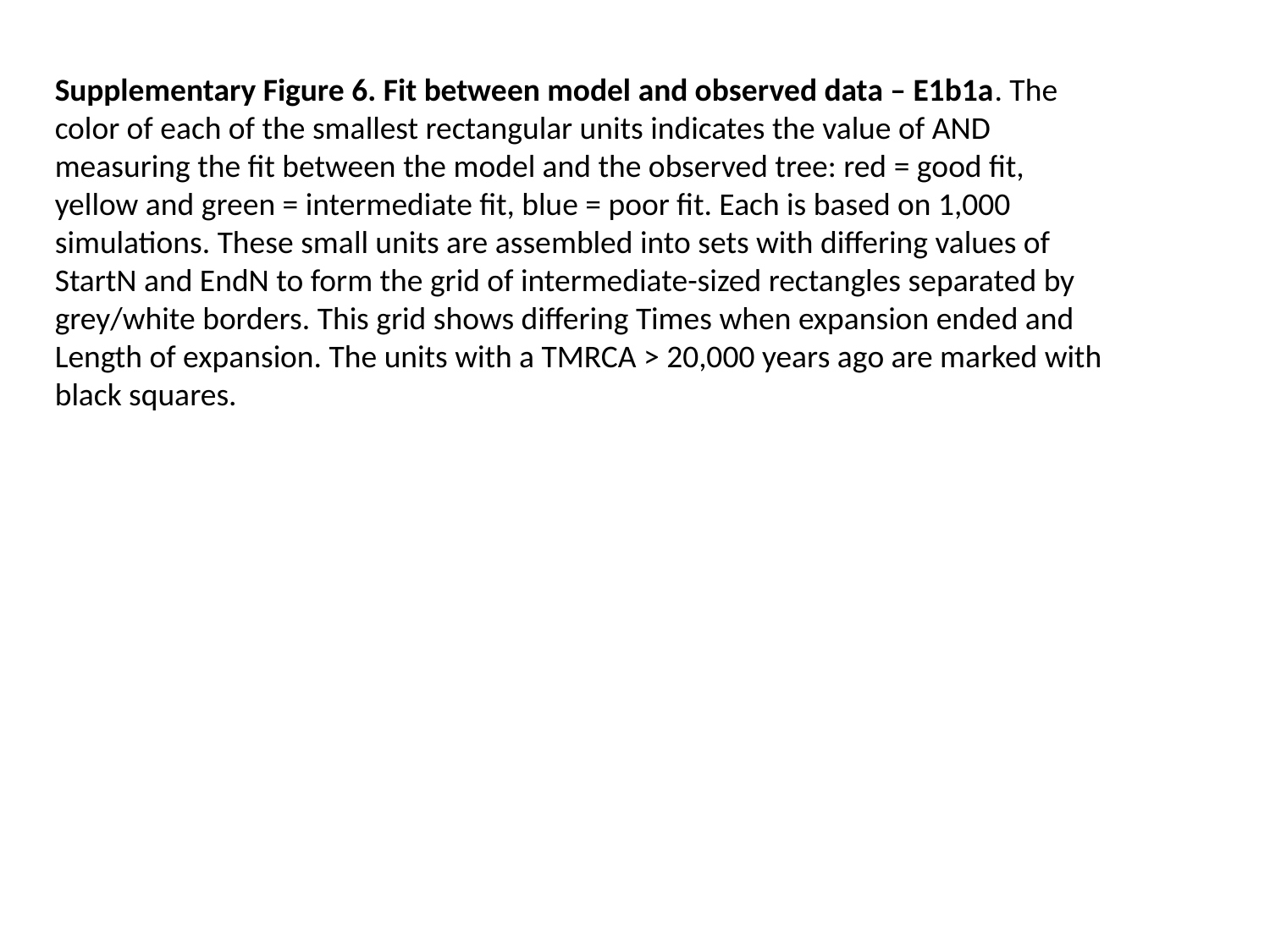

Supplementary Figure 6. Fit between model and observed data – E1b1a. The color of each of the smallest rectangular units indicates the value of AND measuring the fit between the model and the observed tree: red = good fit, yellow and green = intermediate fit, blue = poor fit. Each is based on 1,000 simulations. These small units are assembled into sets with differing values of StartN and EndN to form the grid of intermediate-sized rectangles separated by grey/white borders. This grid shows differing Times when expansion ended and Length of expansion. The units with a TMRCA > 20,000 years ago are marked with black squares.

## Slide 13
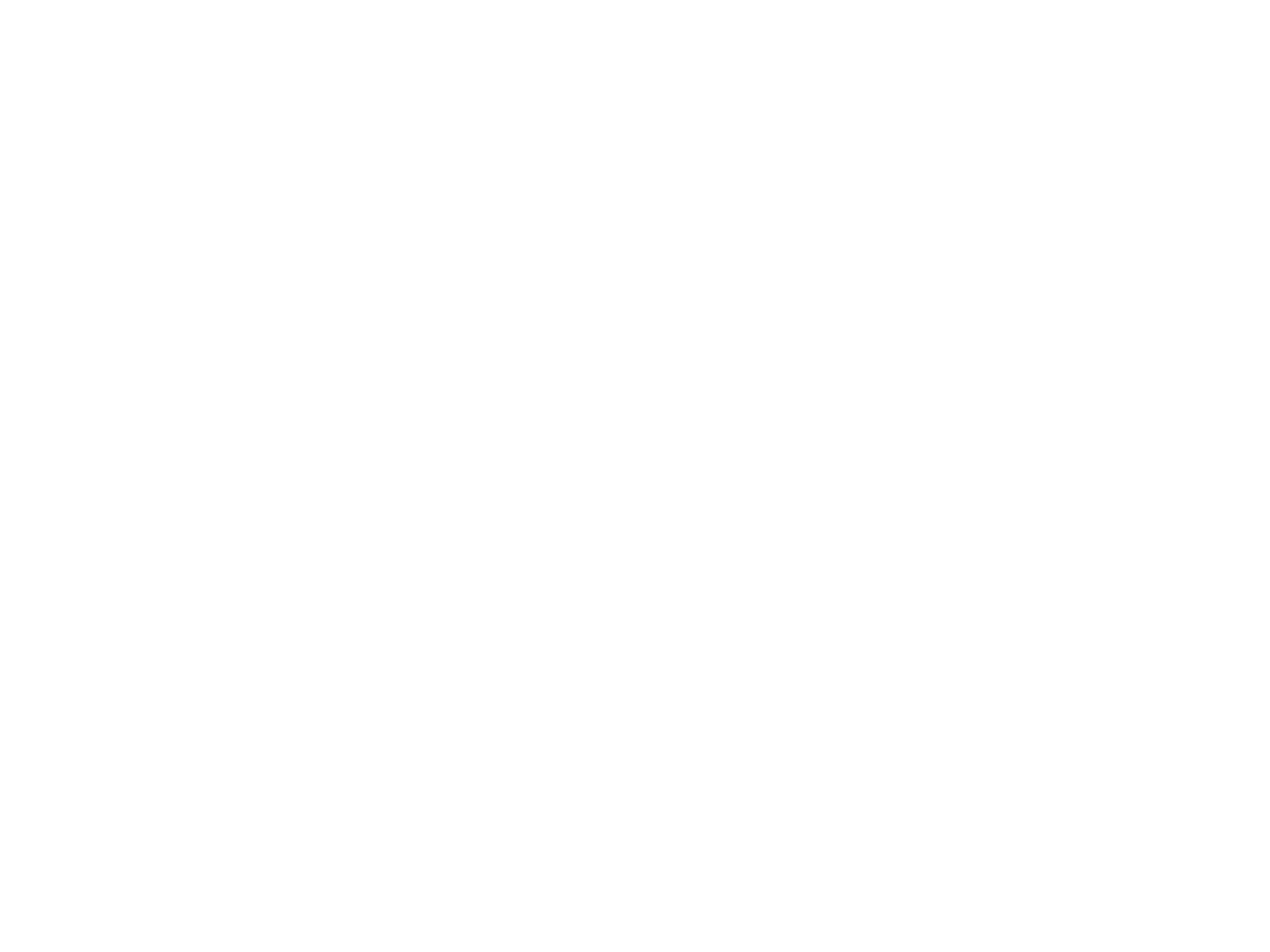

## Slide 14
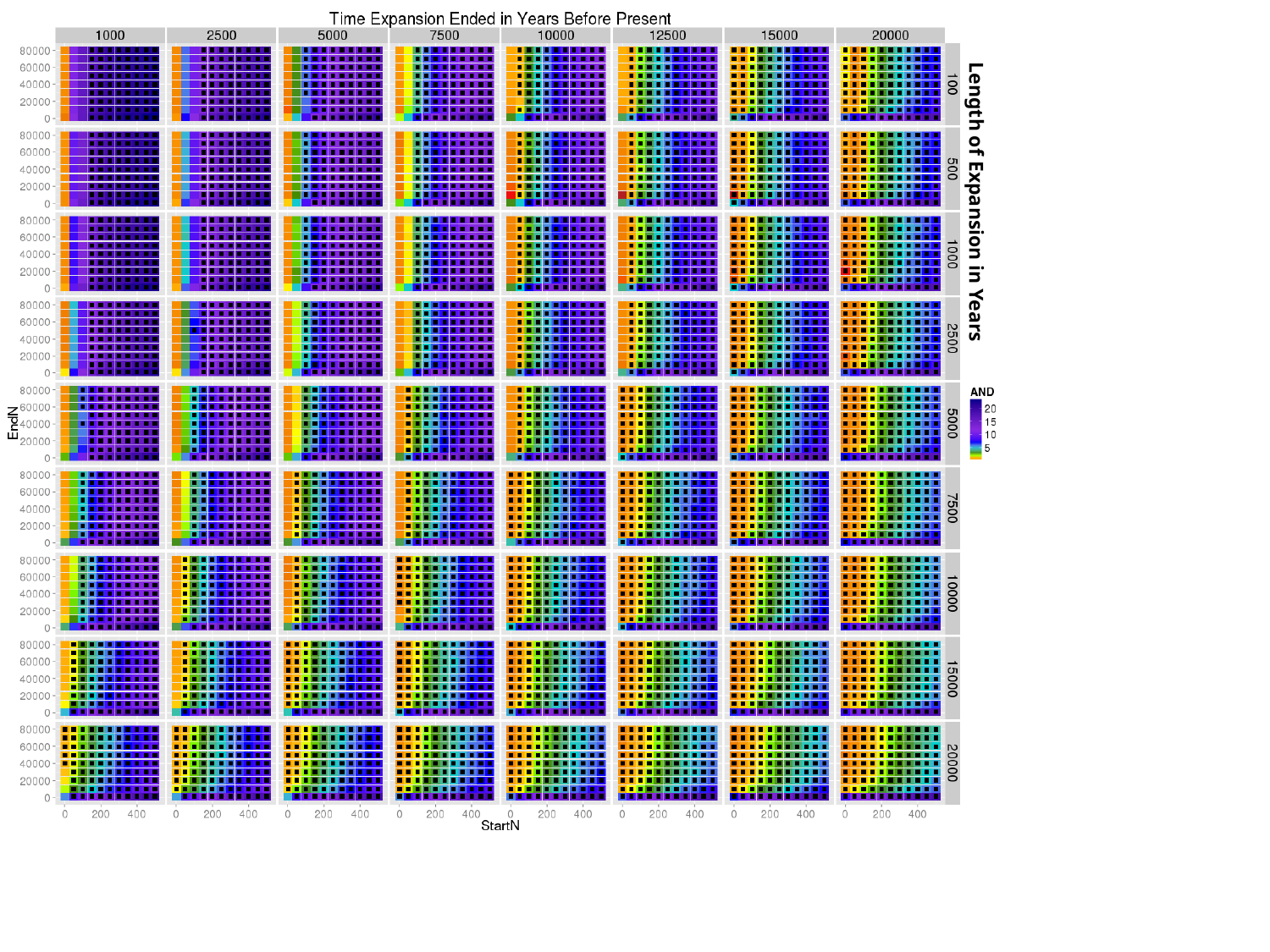

Length of Expansion in Years

## Slide 15
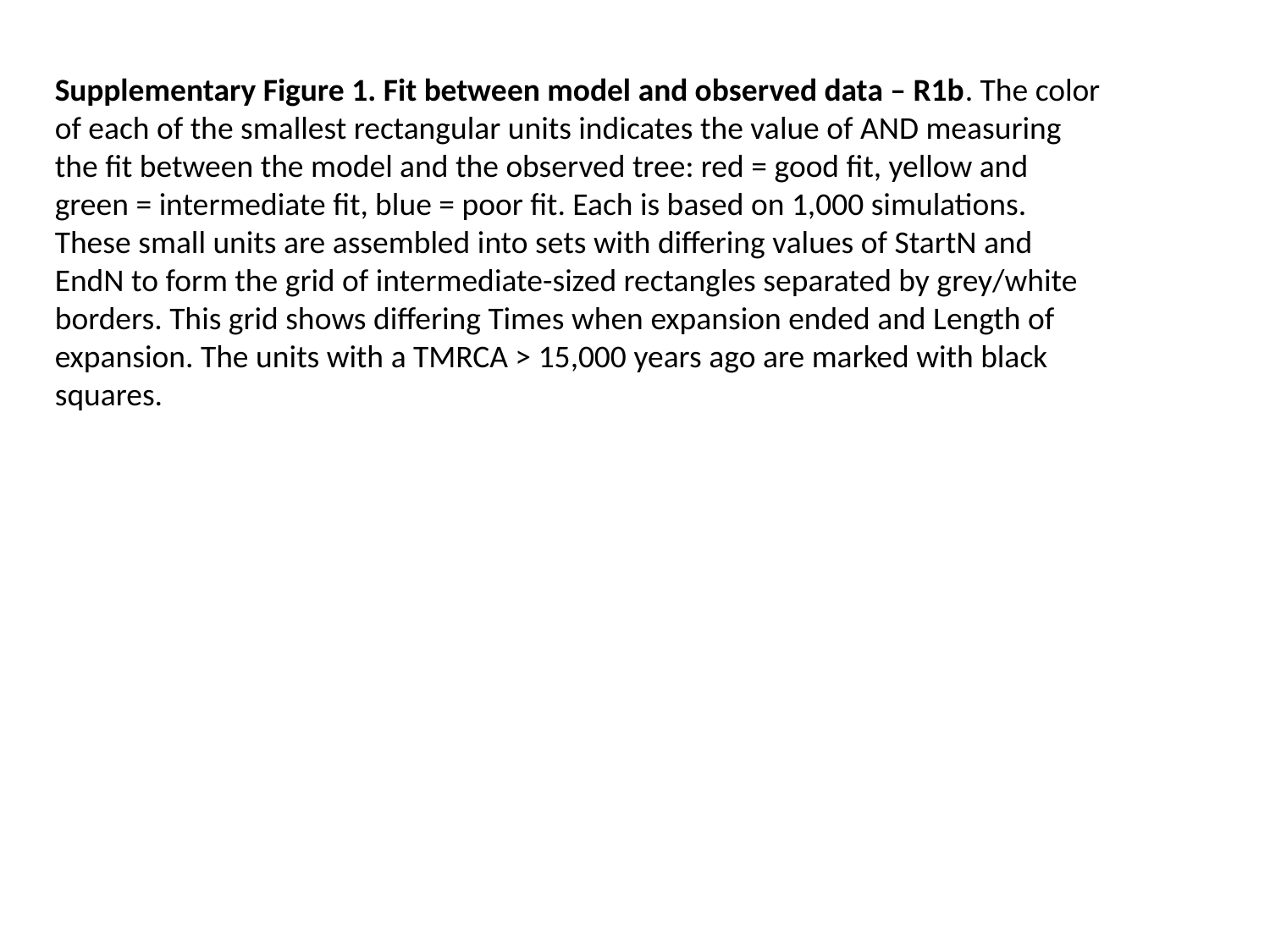

Supplementary Figure 1. Fit between model and observed data – R1b. The color of each of the smallest rectangular units indicates the value of AND measuring the fit between the model and the observed tree: red = good fit, yellow and green = intermediate fit, blue = poor fit. Each is based on 1,000 simulations. These small units are assembled into sets with differing values of StartN and EndN to form the grid of intermediate-sized rectangles separated by grey/white borders. This grid shows differing Times when expansion ended and Length of expansion. The units with a TMRCA > 15,000 years ago are marked with black squares.

## Slide 16
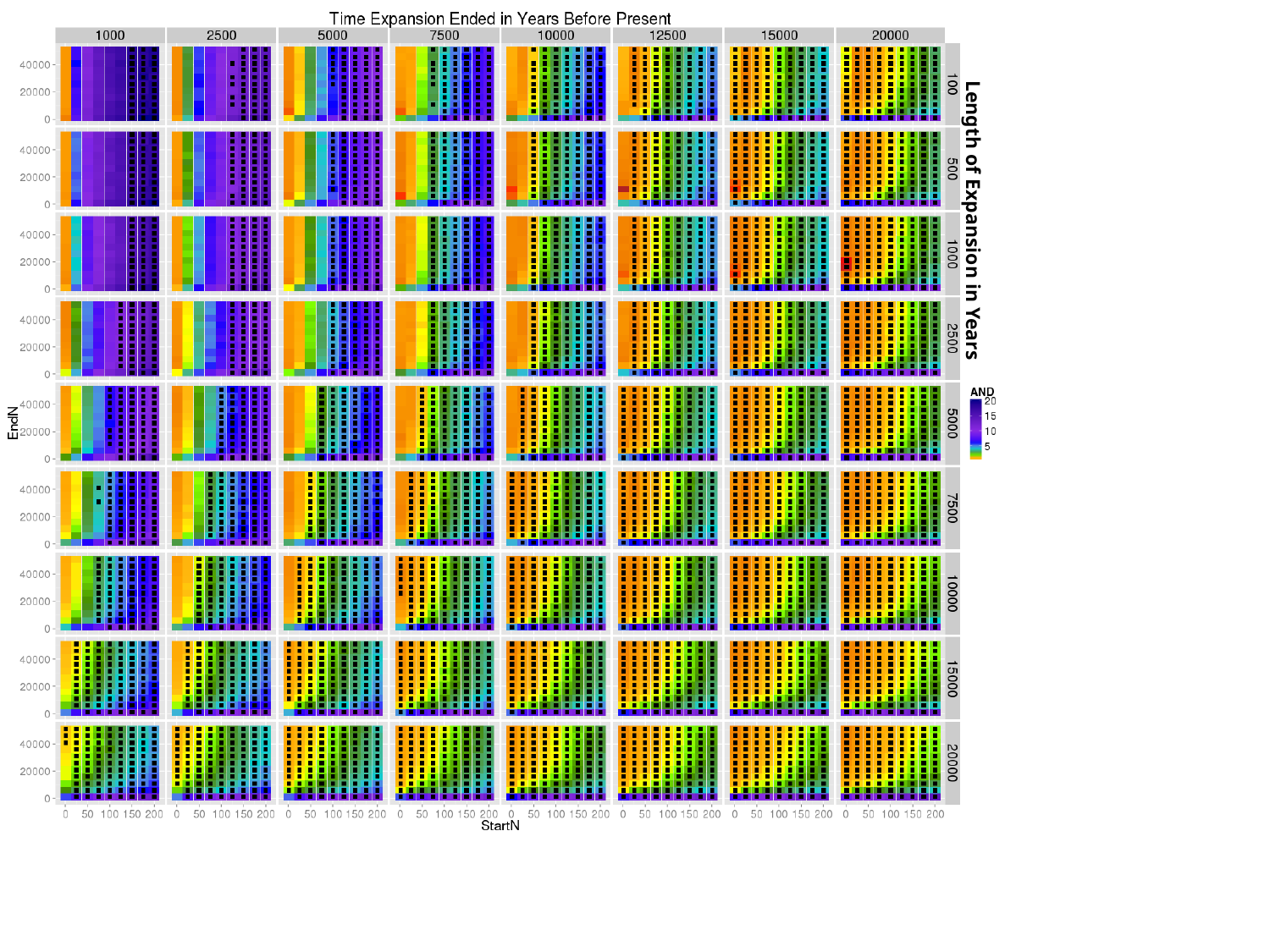

Length of Expansion in Years

## Slide 17
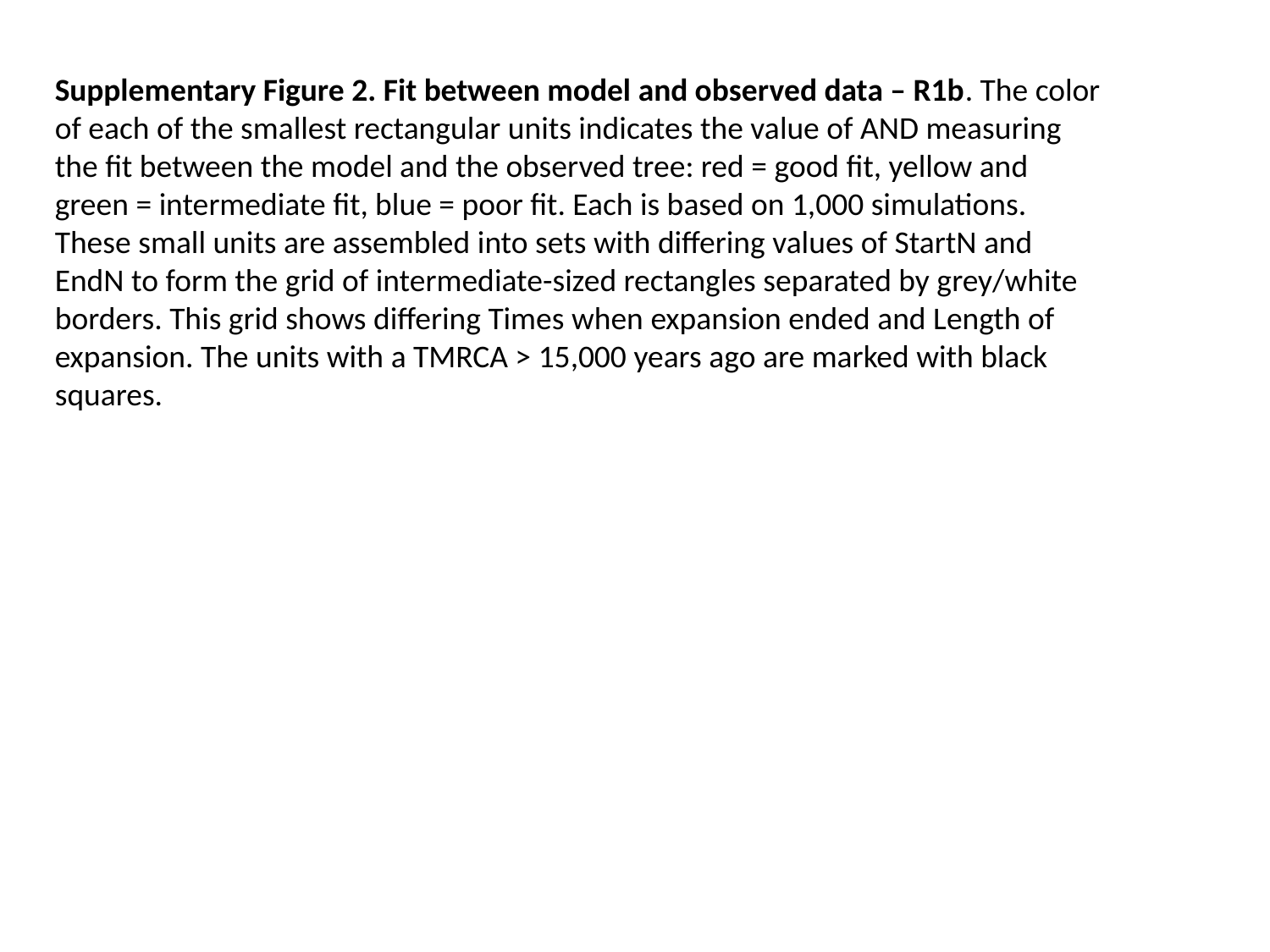

Supplementary Figure 2. Fit between model and observed data – R1b. The color of each of the smallest rectangular units indicates the value of AND measuring the fit between the model and the observed tree: red = good fit, yellow and green = intermediate fit, blue = poor fit. Each is based on 1,000 simulations. These small units are assembled into sets with differing values of StartN and EndN to form the grid of intermediate-sized rectangles separated by grey/white borders. This grid shows differing Times when expansion ended and Length of expansion. The units with a TMRCA > 15,000 years ago are marked with black squares.

## Slide 18
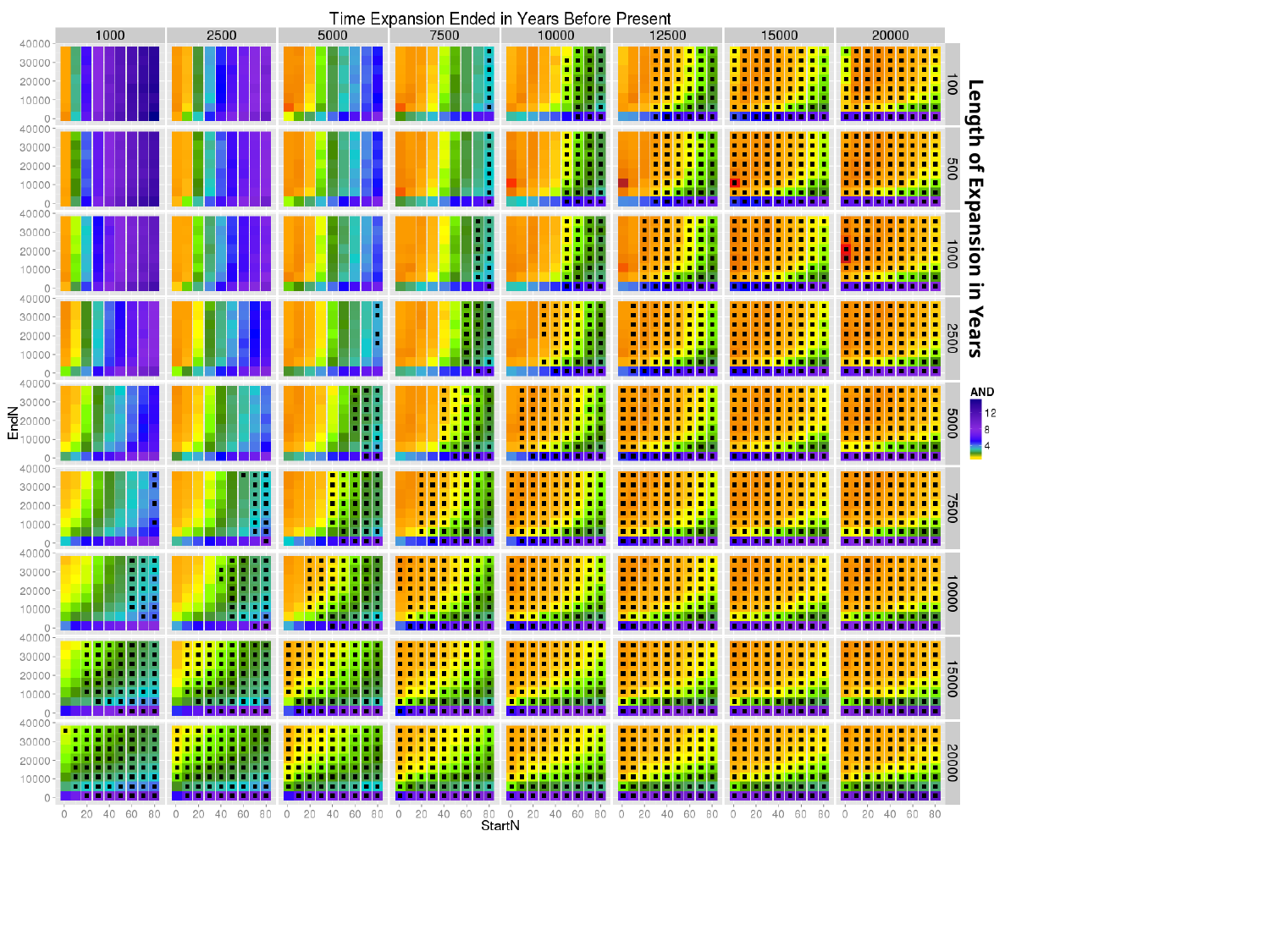

Length of Expansion in Years

## Slide 19
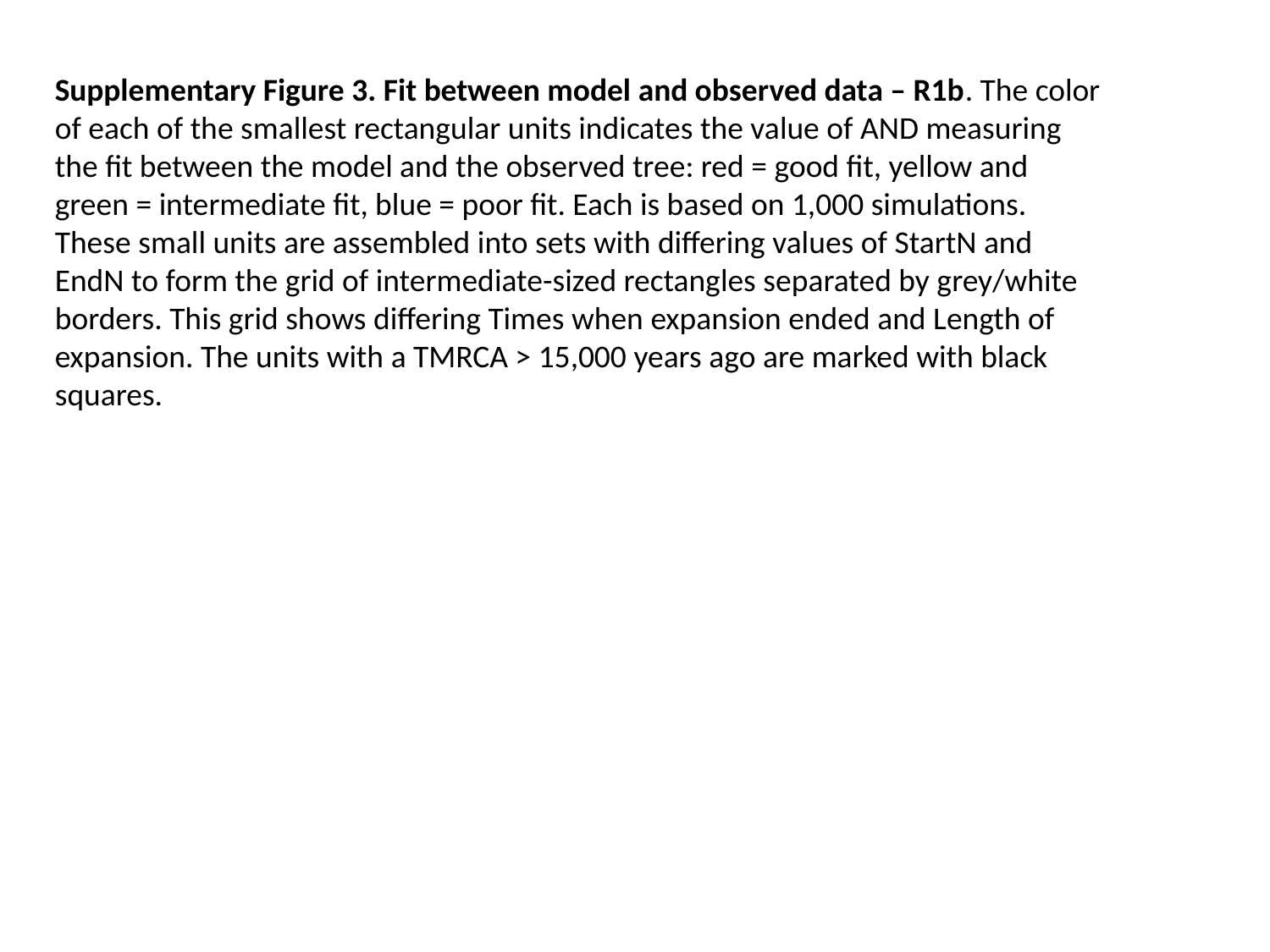

Supplementary Figure 3. Fit between model and observed data – R1b. The color of each of the smallest rectangular units indicates the value of AND measuring the fit between the model and the observed tree: red = good fit, yellow and green = intermediate fit, blue = poor fit. Each is based on 1,000 simulations. These small units are assembled into sets with differing values of StartN and EndN to form the grid of intermediate-sized rectangles separated by grey/white borders. This grid shows differing Times when expansion ended and Length of expansion. The units with a TMRCA > 15,000 years ago are marked with black squares.

## Slide 20
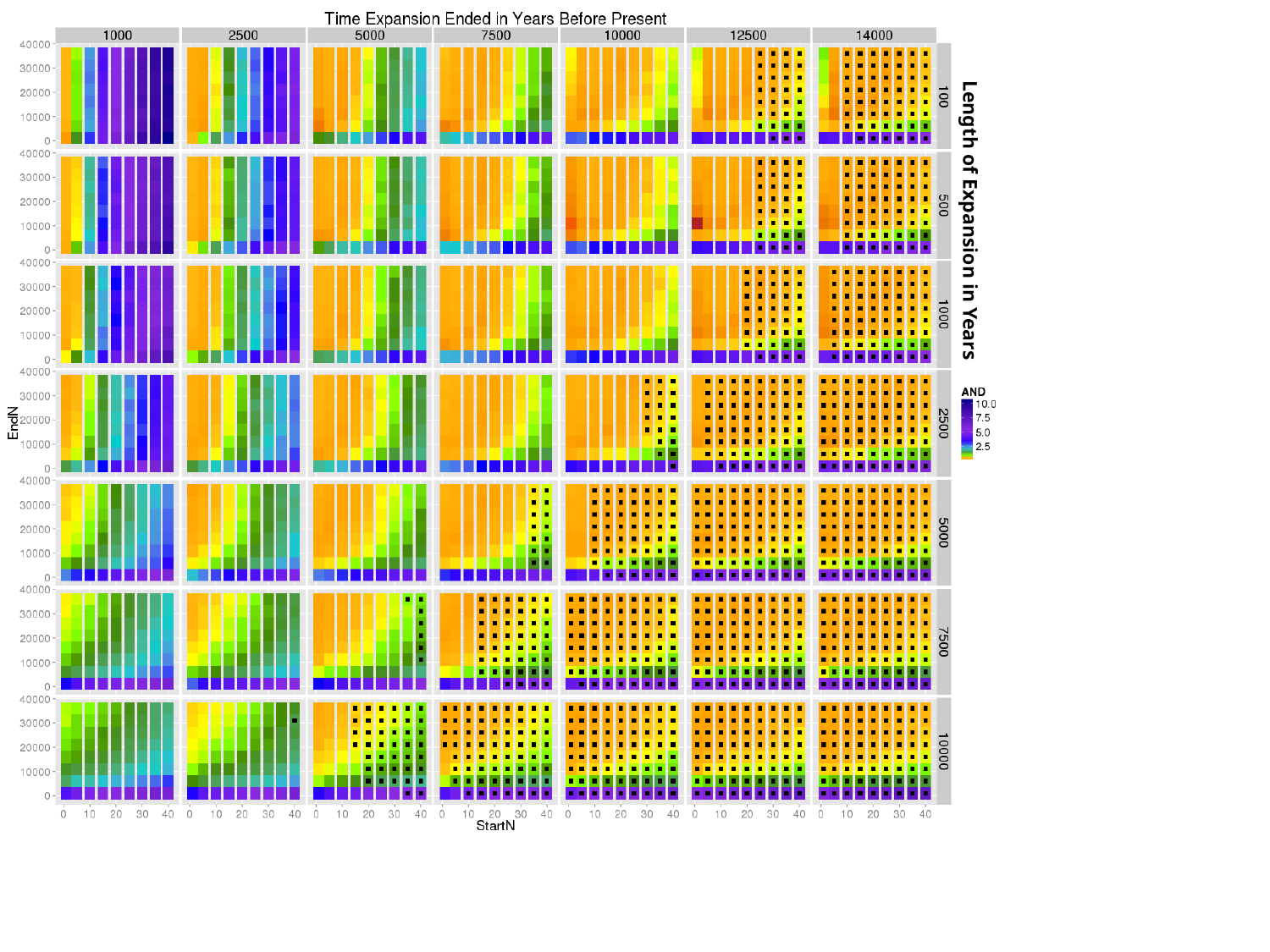

Length of Expansion in Years

## Slide 21
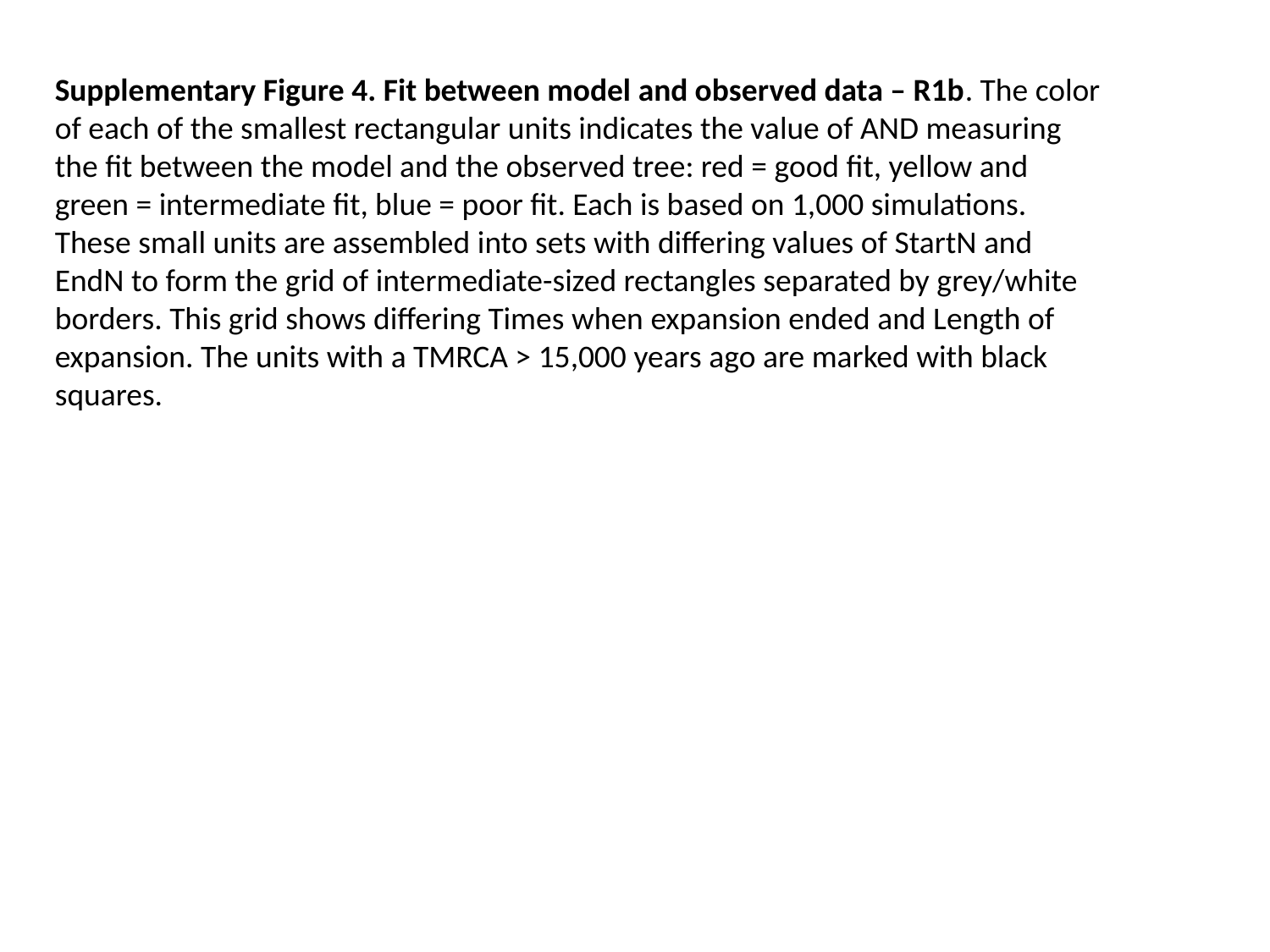

Supplementary Figure 4. Fit between model and observed data – R1b. The color of each of the smallest rectangular units indicates the value of AND measuring the fit between the model and the observed tree: red = good fit, yellow and green = intermediate fit, blue = poor fit. Each is based on 1,000 simulations. These small units are assembled into sets with differing values of StartN and EndN to form the grid of intermediate-sized rectangles separated by grey/white borders. This grid shows differing Times when expansion ended and Length of expansion. The units with a TMRCA > 15,000 years ago are marked with black squares.

## Slide 22
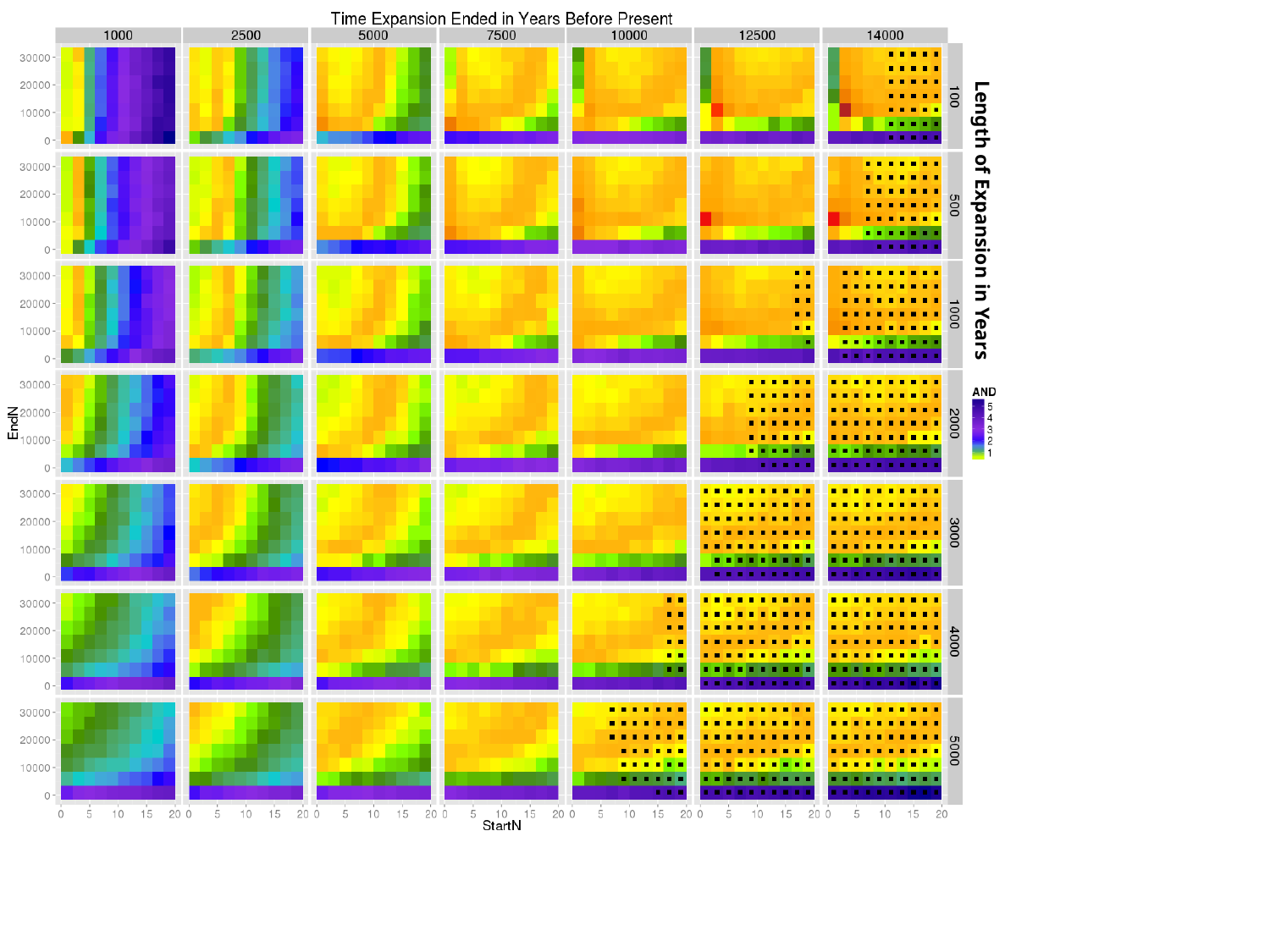

Length of Expansion in Years

## Slide 23
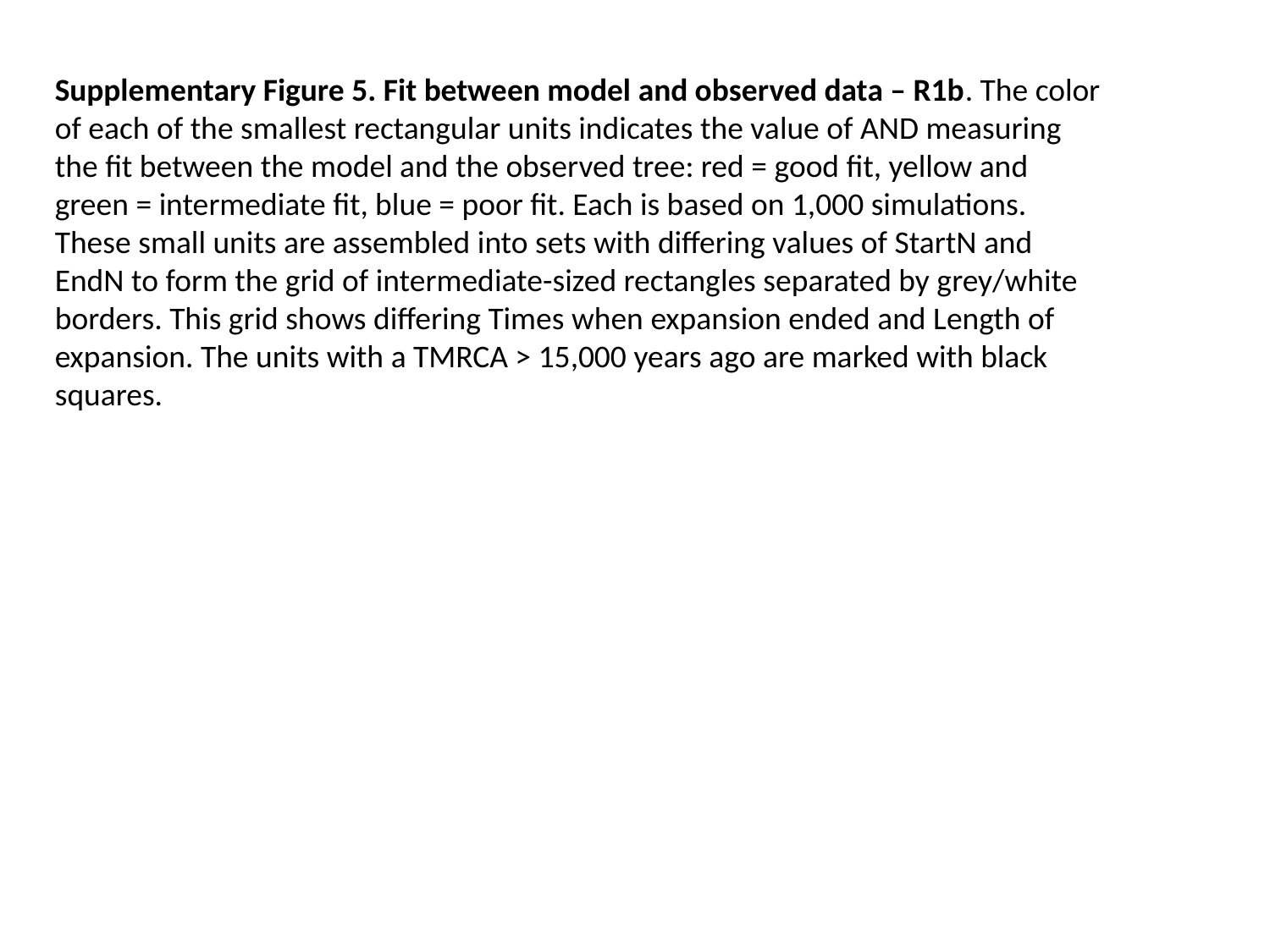

Supplementary Figure 5. Fit between model and observed data – R1b. The color of each of the smallest rectangular units indicates the value of AND measuring the fit between the model and the observed tree: red = good fit, yellow and green = intermediate fit, blue = poor fit. Each is based on 1,000 simulations. These small units are assembled into sets with differing values of StartN and EndN to form the grid of intermediate-sized rectangles separated by grey/white borders. This grid shows differing Times when expansion ended and Length of expansion. The units with a TMRCA > 15,000 years ago are marked with black squares.

## Slide 24
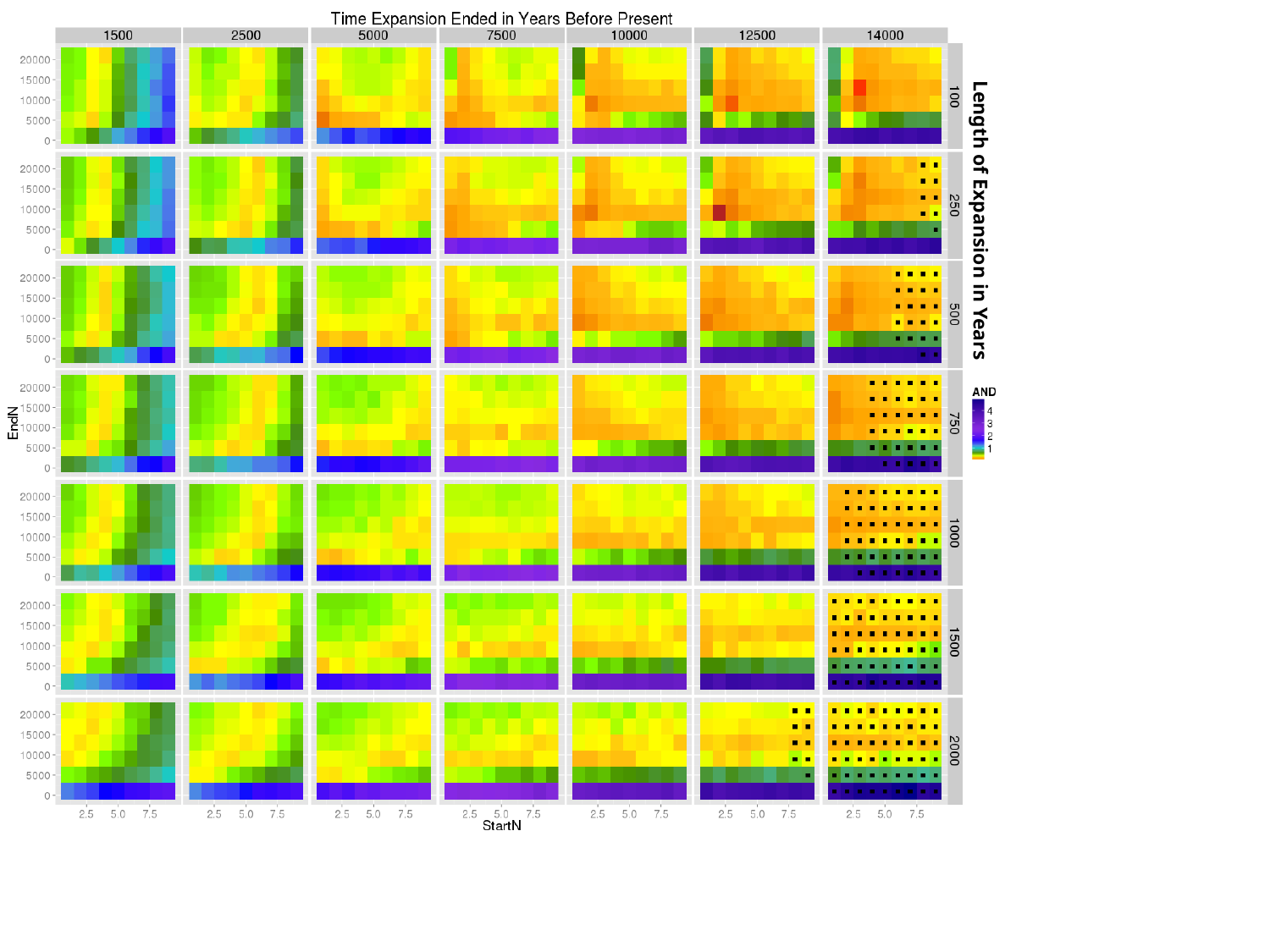

Length of Expansion in Years

## Slide 25
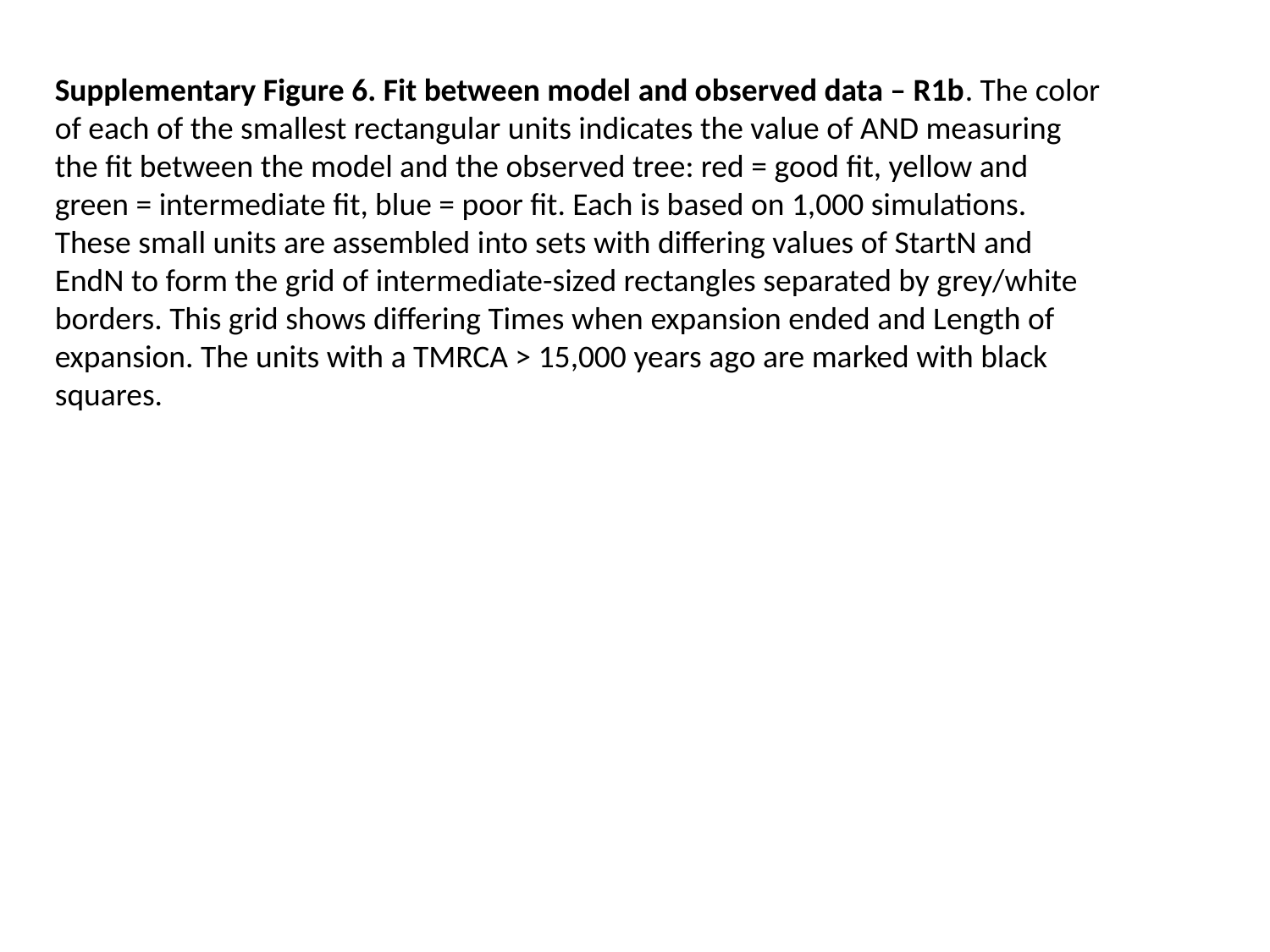

Supplementary Figure 6. Fit between model and observed data – R1b. The color of each of the smallest rectangular units indicates the value of AND measuring the fit between the model and the observed tree: red = good fit, yellow and green = intermediate fit, blue = poor fit. Each is based on 1,000 simulations. These small units are assembled into sets with differing values of StartN and EndN to form the grid of intermediate-sized rectangles separated by grey/white borders. This grid shows differing Times when expansion ended and Length of expansion. The units with a TMRCA > 15,000 years ago are marked with black squares.

## Slide 26
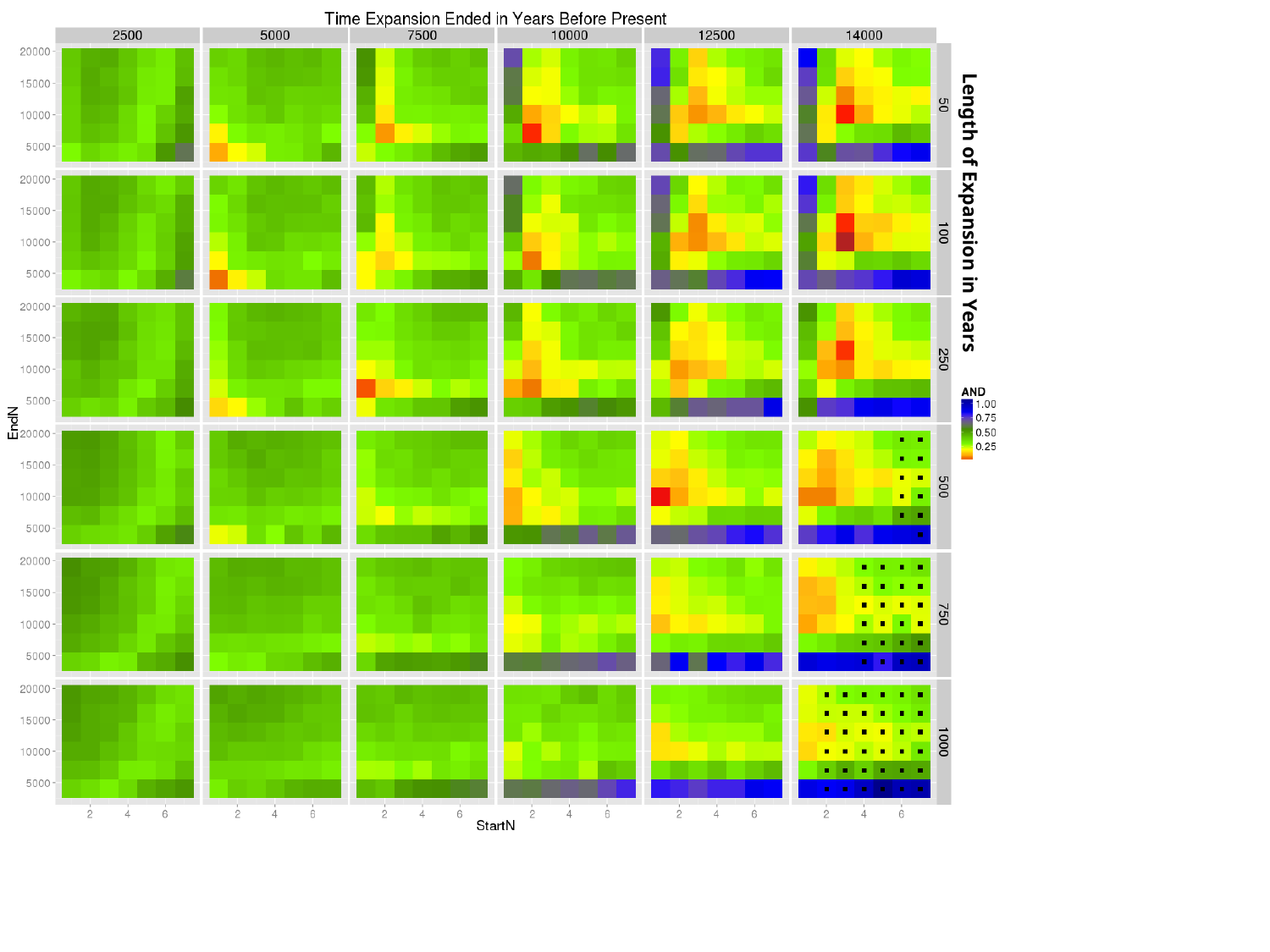

Length of Expansion in Years

## Slide 27
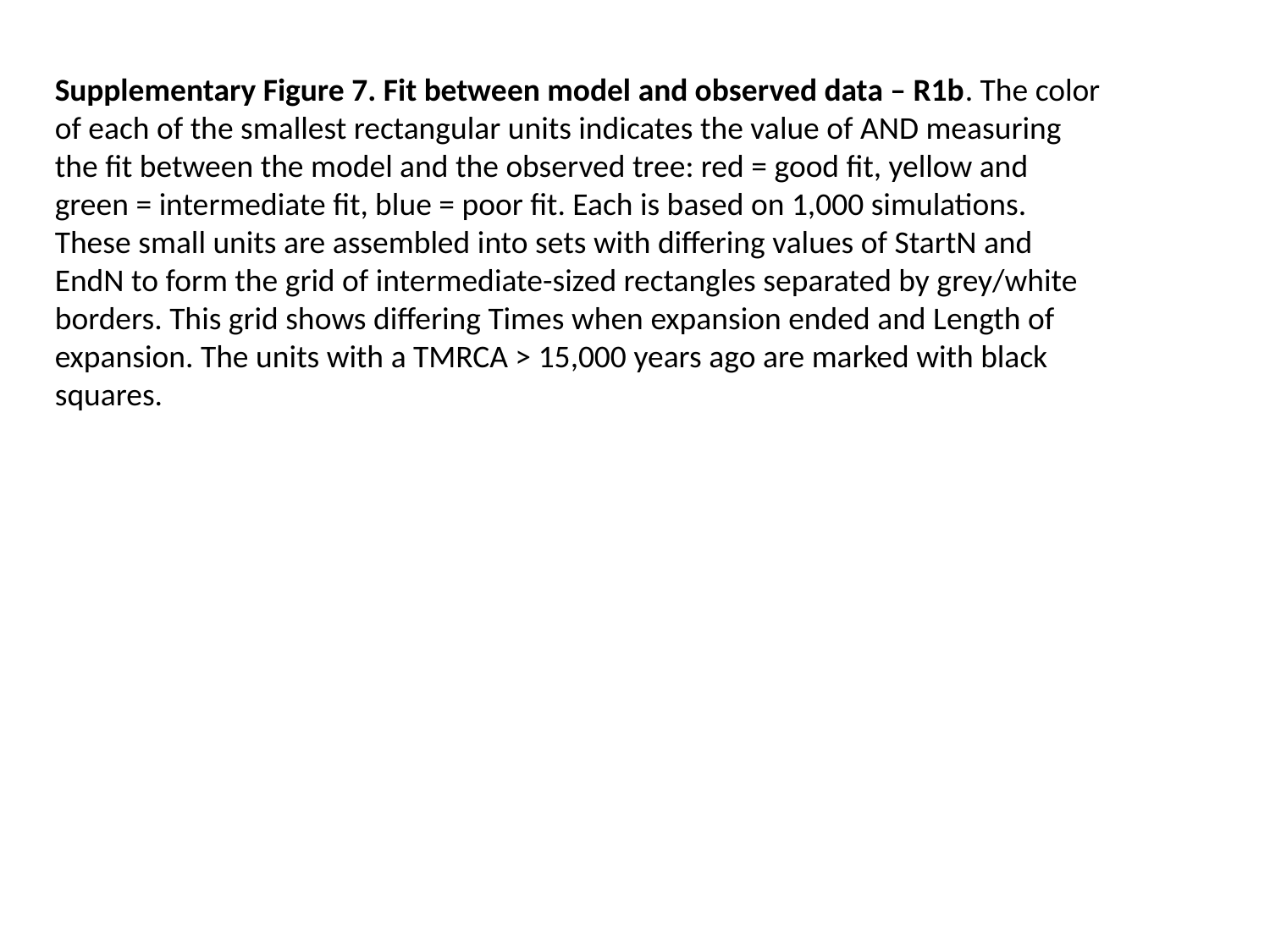

Supplementary Figure 7. Fit between model and observed data – R1b. The color of each of the smallest rectangular units indicates the value of AND measuring the fit between the model and the observed tree: red = good fit, yellow and green = intermediate fit, blue = poor fit. Each is based on 1,000 simulations. These small units are assembled into sets with differing values of StartN and EndN to form the grid of intermediate-sized rectangles separated by grey/white borders. This grid shows differing Times when expansion ended and Length of expansion. The units with a TMRCA > 15,000 years ago are marked with black squares.

## Slide 28
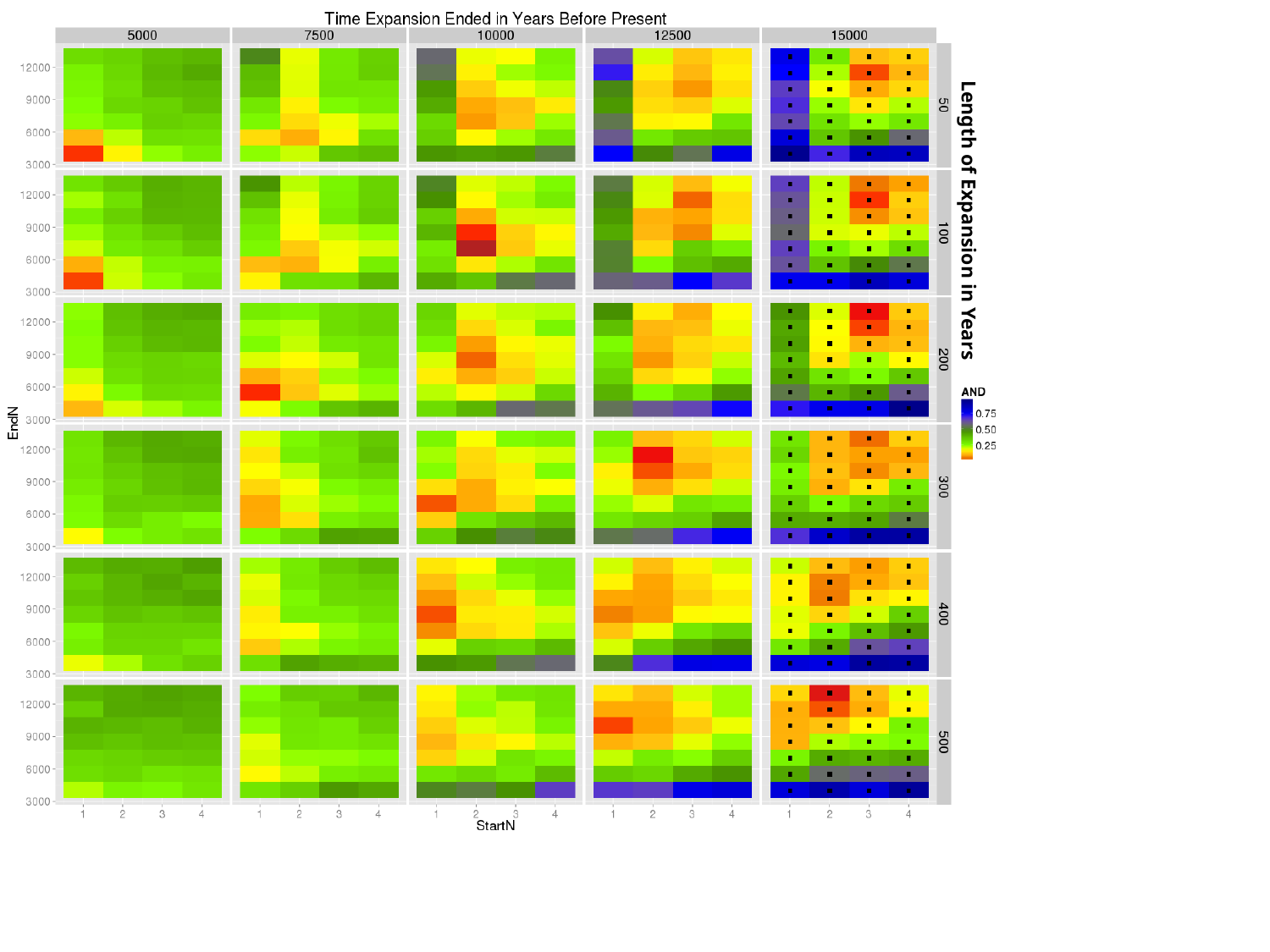

Length of Expansion in Years

## Slide 29
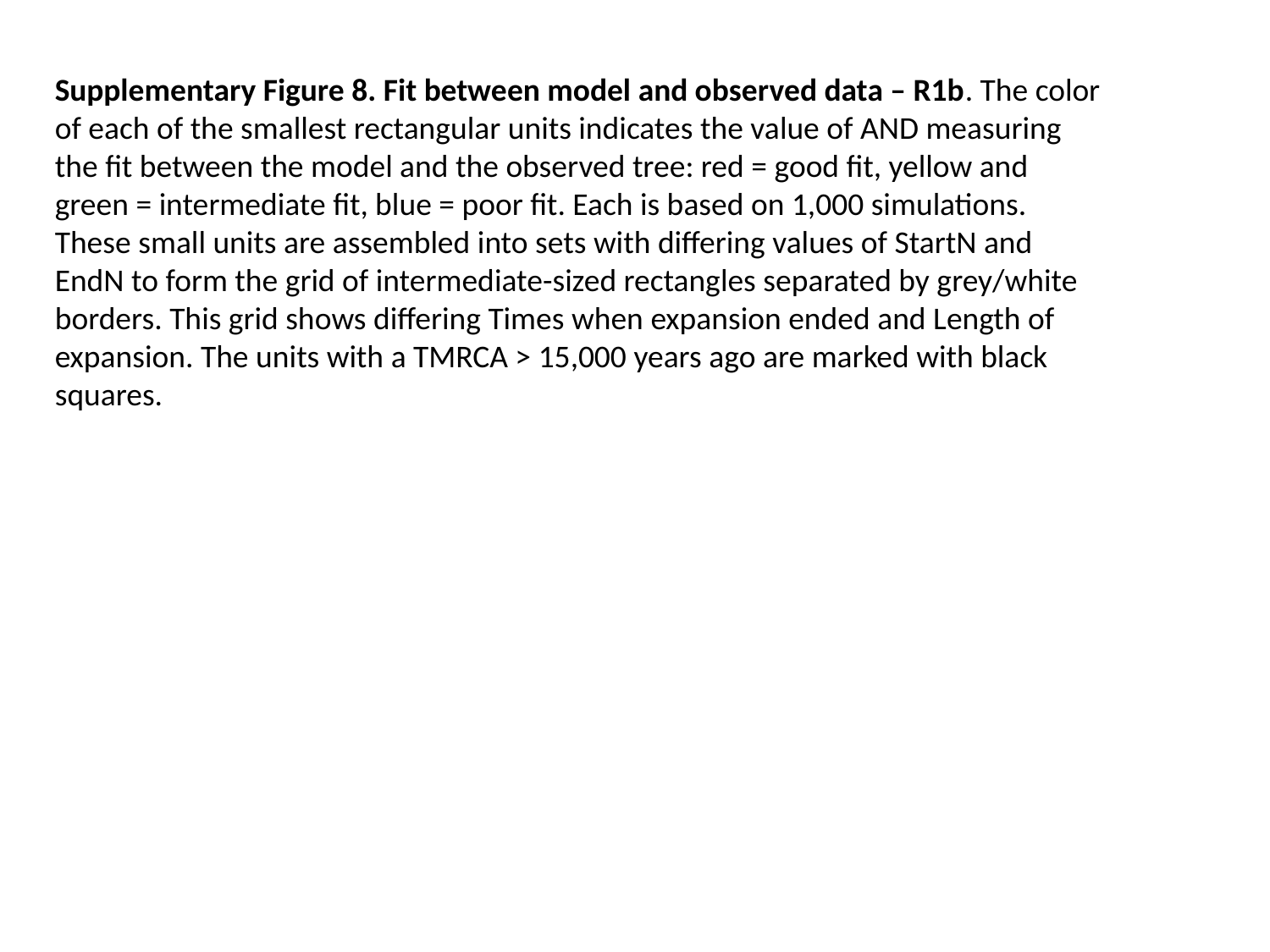

Supplementary Figure 8. Fit between model and observed data – R1b. The color of each of the smallest rectangular units indicates the value of AND measuring the fit between the model and the observed tree: red = good fit, yellow and green = intermediate fit, blue = poor fit. Each is based on 1,000 simulations. These small units are assembled into sets with differing values of StartN and EndN to form the grid of intermediate-sized rectangles separated by grey/white borders. This grid shows differing Times when expansion ended and Length of expansion. The units with a TMRCA > 15,000 years ago are marked with black squares.
